# Disruption of gene *SPL35*, encoding a novel CUE domain‐containing protein, leads to cell death and enhanced disease response in rice

**DOI:** 10.1111/pbi.13093

**Published:** 2019-03-05

**Authors:** Jian Ma, Yongfei Wang, Xiaoding Ma, Lingzhi Meng, Ruonan Jing, Fan Wang, Shuai Wang, Zhijun Cheng, Xin Zhang, Ling Jiang, Jiulin Wang, Jie Wang, Zhichao Zhao, Xiuping Guo, Qibing Lin, Fuqing Wu, Shanshan Zhu, Chuanyin Wu, Yulong Ren, Cailin Lei, Huqu Zhai, Jianmin Wan

**Affiliations:** ^1^ Institute of Crop Sciences Chinese Academy of Agricultural Sciences/National Key Facility for Crop Gene Resources and Genetic Improvement Beijing China; ^2^ Key Laboratory of Crop Genetics and Germplasm Enhancement/Jiangsu Provincial Center of Plant Gene Engineering Nanjing Agricultural University Nanjing China

**Keywords:** *Oryza sativa*, *SPL35*, CUE domain‐containing protein, cell death, disease resistance

## Abstract

Lesion mimic mutants that exhibit spontaneous hypersensitive response (HR)‐like necrotic lesions are ideal experimental systems for elucidating molecular mechanisms involved in plant cell death and defence responses. Here we report identification of a rice lesion mimic mutant, *spotted leaf 35* (*spl35*), and cloning of the causal gene by TAIL‐PCR strategy. *spl35* exhibited decreased chlorophyll content, higher accumulation of H_2_O_2_, up‐regulated expression of defence‐related marker genes, and enhanced resistance to both fungal and bacterial pathogens of rice. The *SPL35* gene encodes a novel CUE (coupling of ubiquitin conjugation to ER degradation) domain‐containing protein that is predominantly localized in cytosol, ER and unknown punctate compartment(s). *SPL35* is constitutively expressed in all organs, and both overexpression and knockdown of *SPL35* cause the lesion mimic phenotype. SPL35 directly interacts with the E2 protein OsUBC5a and the coatomer subunit delta proteins Delta‐COP1 and Delta‐COP2 through the CUE domain, and down‐regulation of these interacting proteins also cause development of HR‐like lesions resembling those in *spl35* and activation of defence responses, indicating that *SPL35* may be involved in the ubiquitination and vesicular trafficking pathways. Our findings provide insight into a role of SPL35 in regulating cell death and defence response in plants.

## Introduction

Plants have evolved various defence mechanisms to protect themselves from pathogen attack, the most common of which is the hypersensitive response (HR) that acts to inhibit continued invasion or proliferation of pathogens in host plant tissues (Fujiwara *et al*., 2010; Wang *et al*., 2017). HR involves bursts of reactive oxygen species (ROS), expression of pathogenesis‐related (PR) genes, accumulation of phytoalexins and cell wall fortification through callose deposition, all of which help to inhibit pathogen proliferation (Shirsekar *et al*., 2014; Wang *et al*., 2017). Although HR plays a crucial role in plant disease resistance, its underlying molecular mechanisms are not fully understood.

To gain insights into HR‐mediated cell death in plants, a number of lesion mimic mutants (LMMs), showing typical HR‐like lesions independent of pathogen infection, have been investigated in a range of plant species, including *Arabidopsis* (Dietrich *et al*., 1994; Lorrain *et al*., 2004), maize (Johal *et al*., 1995; Walbot, 1991) and rice (Wang *et al*., 2015a, 2017). Many LMMs are known to have activated defence responses and sometimes display significantly enhanced resistance to disease, and thus are considered ideal for studying signalling pathways involved in HR‐mediated cell death (Shirsekar *et al*., 2014; Wang *et al*., 2015a,2015b, 2017). Identification and characterization of genes involved in LMMs may facilitate the elucidation of molecular mechanisms underlying plant disease resistance (Shirsekar *et al*., 2014; Wang *et al*., 2015b, 2017).

Over the past two decades, a number of lesion mimic genes have been cloned. These genes encode various proteins involved in numerous defence‐related signalling pathways, including those related to ROS generation, disease resistance, calcium influx, and protein ubiquitination or phosphorylation, as well as biosynthesis or metabolism of fatty acids/lipids, porphyrin and phenolic compounds. The identities of these genes indicated that the LMM phenotype could be the result of not only alternations in the HR‐response to pathogens but also due to changes in various physiological pathways essential in response to both biotic and abiotic stresses (Undan *et al*., 2012; Wang *et al*., 2015b, 2017).

Protein ubiqutination has emerged as a key mechanism involved in growth and development as well as immunity in animals and plants (Liu *et al*., 2017; You *et al*., 2016). Recognition of ubiquitin or specific ubiquitin chains by ubiquitin‐binding domains (UBDs) is vital for deciphering ubiquitin‐mediated signalling pathways (Liu *et al*., 2012a). Among a handful of types of characterized UBDs, the coupling of ubiquitin conjugation to endoplasmic reticulum (ER) degradation (CUE) domains consisting of 42–43 amino acid sequences have been identified and characterized based on similarity to the region of the yeast Cue1 protein, which is implicated in yeast ER‐associated degradation (ERAD; Shih *et al*., 2003); however, their roles in ERAD remain poorly understood (Li *et al*., 2017; Liu *et al*., 2012a; Shideler *et al*., 2015). In animals, some CUE domain‐containing (CUEDC) proteins have been implicated in programmed cell death (PCD) and immunity. For examples, the CUEDC protein, ERAD E3 gp78, is a regulator of liver homeostasis and a tumour suppressor in liver, and its CUE domain interacts with ubiquitin and diubiquitin and functions to facilitate substrate binding and processivity in ubiquitination (Liu *et al*., 2012a; Zhang *et al*., 2015); another CUEDC protein, CUEDC2, suppresses glioma tumorigenicity by inhibiting the activation of STAT3 and NF‐κB, and its CUE domain is essential for functional interactions with both monoubiquitin and polyubiquitin (Li *et al*., 2017). In plants, the reported CUECD proteins are the duplicated pair of *Arabidopsis* RING‐finger E3 ligases, RIN2 and RIN3, which each possess a RING‐finger domain in association with a CUE domain; however, the CUE domain was rarely characterized for its roles in the E3 ligases (Kawasaki *et al*., 2005). Therefore, the question whether CUEDC proteins are involved in immunity and ubiquitination pathways in plants remains to be answered.

In the present study, we isolated and characterized a rice LMM named *spotted leaf 35* (*spl35*) from a T‐DNA insertion population of cultivar (cv.) Kinmaze. *spl35* displayed spotted leaves and enhanced resistance to rice blast and bacterial blight. We cloned *SPL35* by TAIL‐PCR (thermal asymmetric interlaced PCR) strategy. *SPL35* encodes a novel CUEDC protein and is expressed at all developmental stages and in all tissues examined. SPL35 is mainly localized in cytosol, ER and unknown compartment(s). SPL35 directly interacts with an E2 protein, OsUBC5a, and two coatomer subunit delta proteins, Delta‐COP1 and Delta‐COP2 both *in vitro* and *in vivo*. Our results indicate that SPL35 is involved in ubiquitination and vesicular trafficking, and disruption of *SPL35* can result in cell death and enhanced defence response in rice.

## Results

### Phenotypic characteristics of the *spl35* mutant

**Figure 1:**
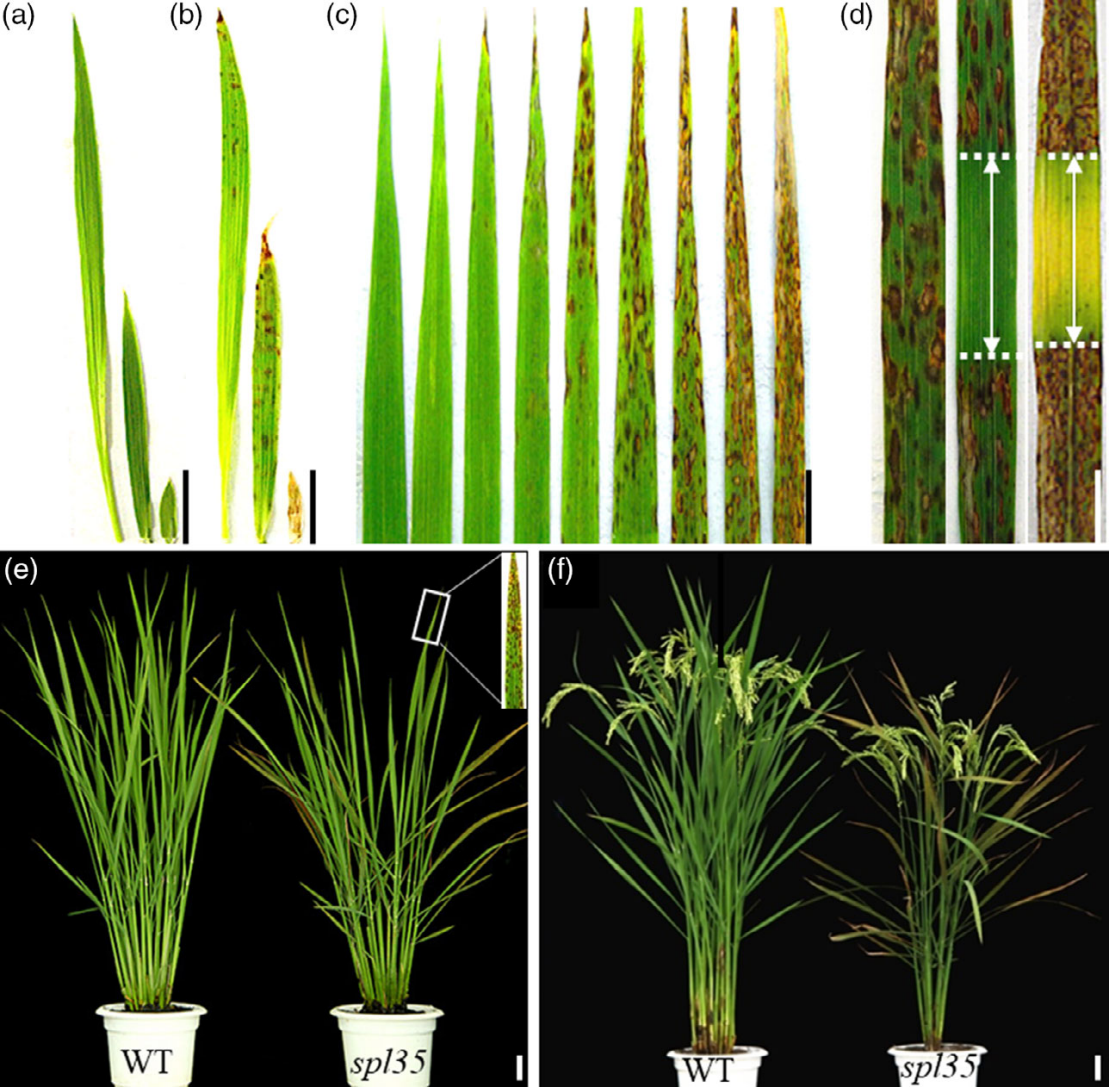
Phenotypes of the *spotted leaf 35* (*spl35*) mutant. (a and b) Leaf blade phenotypes of wild‐type (WT) (a) and *spl35* (b) at four‐leaf stage; left to right: fourth, third and second leaf blades respectively. (c) Lesion mimic phenotypes of different leaves in the main culm of *spl35* plants at the tillering stage corresponding to (e). From left to right: youngest leaf to oldest leaf blades respectively. (d) Light avoidance assay of lesion mimic flag leaves. Left: leaf blade with no light avoidance; middle and right: leaf blade wrapped with aluminium foil in the middle part for 7 and 14 days respectively. (e) Plants at tillering stage (60 days after seed sowing) in the paddy field. (f) Plants at 15 days after flowering in the paddy field. *Scale bars*, 5.0 cm.

We isolated a LMM, named *spotted leaf 35* (*spl35*), from a T‐DNA insertion population created by transformation of cv. Kinmaze. The *spl35* plants began to show small, reddish‐brown lesions or spots from top to bottom of leaves at the three‐leaf stage [about 20 days after seed sowing (DAS)], and such spots gradually developed and scattered on every fully expanded leaf throughout the life cycle (Figure 1a–c). In general, young leaves displayed fewer and smaller lesions, and old leaves had more and larger lesions that frequently merged into each other, in contrast to green healthier leaves in wild‐type (WT, Kinmaze; Figure 1c). As a result, *spl35* had significant reduction in plant height, number of effective panicles, number of spikelets per panicle, seed setting rate and 1000‐grain weight by 11.39%, 21.06%, 24.88%, 20.31% and 9.30%, respectively, compared to WT, but no change in heading date and panicle length (Figure 1e,f; Table S2).

It is often seen that development of lesions in LMMs is associated with exposure to light (Wang *et al*., 2017). Thus, we performed light avoidance assays on *spl35* as described previously (Wang *et al*., 2017). The leaf area without exposure to light did not develop lesions of any size, whereas the neighbouring uncovered area did develop numerous lesions (Figure 1d), indicating that formation of lesions in *spl35* was light‐dependent.

To examine physiological changes in *spl35*, we measured chlorophyll (Chl a and Chl b) and carotenoid contents in flag leaves of field‐grown *spl35* and WT plants at 10 days after flowering (DAF). Both chlorophyll and carotenoid contents in *spl35* were significantly decreased relative to the WT (Figure S1a). We further investigated chloroplast development in *spl35* and WT leaves at 10 DAF by TEM. The chloroplast in the spotted leaf area of *spl35* was abnormally shaped, with destructed thylakoid stacking, in contrast to elliptical shape of WT chloroplasts with neatly stacked thylakoids and normal grana (Figure S1b,c). Thus, degradation of thylakoids in damaged chloroplasts of *spl35* impaired photosynthesis capacity and in turn lead to smaller plant size.

### Cell death and H_2_O_2_ accumulation in the *spl35* mutant

To identify possible biochemical mechanisms underlying the development of HR‐like lesions in *spl35*, we investigated expression of histochemical markers for cell death and H_2_O_2_, in *spl35* and WT. Trypan blue staining, a method to detect cell death (Dietrich *et al*., 1994), showed a number of deep blue spots at lesion sites in *spl35*, but not in WT (Figure S2a). H_2_O_2_ is a membrane‐permeable ROS and one of the major signalling molecules causing cellular damage or triggering PCD (Wang *et al*., 2017). We examined H_2_O_2_ level by 3,3′‐diaminobenzidine (DAB) staining and found consistently higher H_2_O_2_ accumulation in *spl35* than in WT (Figure S2b). In addition, the H_2_O_2_ contents in *spl35* seedlings at the two‐ to three‐leaf (before appearance of visible lesions) and at the four‐ to five‐leaf stage (lesions clearly visible) were approximately 1.5‐ and 2‐fold higher than in WT leaves respectively (Figure S2c). These results suggest that the formation of mimic lesions was associated with ROS accumulation and irreversible membrane damage in the *spl35* cells.

### Activation of defence responses in the *spl35* mutant

**Figure 2:**
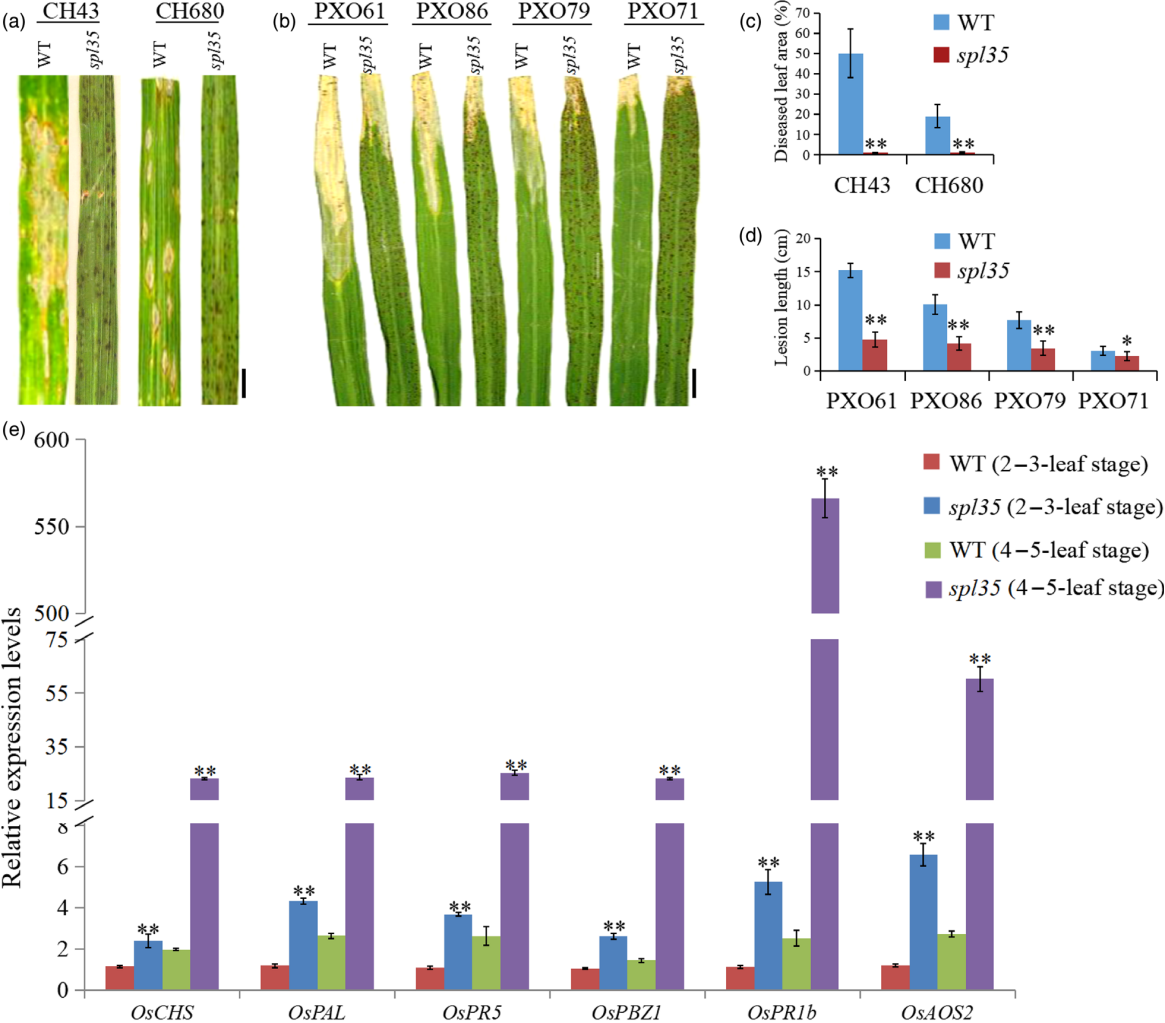
Resistance reactions to *Maganaporthe oryzae* (*M. oryzae*) and *Xanthomonas oryzae* pv*. oryzae* (*Xoo*) isolates tested and expression analysis of defence‐related markers in wild‐type (WT) and *spl35* plants. (a and b) Disease symptoms of WT and *spl35* leaves after challenge with *M. oryzae* (a) and *Xoo* (b) isolates. (c) Diseased leaf area of WT and *spl35* leaves scanned at 7 days after inoculation with two different *M. oryzae* isolates using the Chalkiness 1.0 software (Zheng and Wu, 2008). Asterisks indicate significant differences between WT and *spl35* leaves (**P < 0.01; Student's *t* test). Data are means ± SD of at least 10 leaves. (d) Mean lesion length of WT and *spl35* leaves measured at 14 days after inoculation with four different *Xoo* isolates. Asterisks indicate significant differences between WT and *spl35* mutant plants (*P < 0.05, **P < 0.01; Student's *t* test). Data are means ± SD of at least 10 leaves. (e) qRT‐PCR analysis of defence‐related markers in WT and *spl35* mutant at two‐ to three‐leaf stage (before appearance of visible lesions in *spl35*) and four‐ to five‐leaf stage (lesions clearly visible in *spl35*) respectively. Similar results were obtained in three independent biological replicates. Asterisks indicate significant differences between WT and *spl35* seedlings (**P < 0.01; Student's *t* test). *Scale bars*, 2.0 cm.

In some LMMs, activation of defence responses and enhanced disease resistance have been reported (Tang *et al*., 2011). We first analysed the expression level of ER chaperone genes including *OsCNX1*,* OsBip1* and *OsPDIL1‐1* that are known indicators of accumulation of unfolded proteins in ER caused by accumulation of ROS (Cui *et al*., 2012a,2012b), and found that the RNA expression level of these genes was increased by 1.8‐, 1.5‐ and 2.3‐folds, respectively, in *spl35* relative to WT (Figure S3). Then, we investigated resistance of *spl35* to blast and bacterial blight diseases with isolates of two *Maganaporthe oryzae* (*M. oryzae*) and four *Xanthomonas oryzae* pv*. oryzae* (*Xoo*) pathotypes virulent to WT at the tillering stage, and observed significantly gained disease resistance in *spl35* (Figure 2a–d).

In order to understand the relation of the gained resistance with the development of HR‐like lesions in *spl33*, we inoculated *spl35*, WT and LTH (a variety susceptible to a wide range of isolates) seedlings at the two‐ to three‐leaf and four‐ to five‐leaf stages with isolates of six *M. oryzae* pathotypes virulent to WT. The *spl35* seedlings infected at the four‐ to five‐leaf stage exhibited blast resistance as indicated by lack of newly developed lesions, whereas those infected at the two‐ to three‐leaf stage exhibited fewer and smaller blast lesions than WT and LTH, a sign of moderate resistance (Figure S4; Table S3). This observation suggests that the defence response was initiated before the appearance of the visible mimic lesions in *spl35*.

We then examined the expression of six defence‐related (DR) or pathogenesis‐related (PR) genes, *OsCHS*,* OsPAL*,* OsPR5*,* OsPBZ1*,* OsPR1b* and *OsAOS2* (Liu *et al*., 2012b; Tang *et al*., 2011), in *spl35* and WT at two‐ to three‐leaf stages and four‐ to five‐leaf stages by qRT‐PCR. At the two‐ to three‐leaf stage expression level of these six DR/PR genes in *spl35* was up‐regulated by 2.1‐, 3.6‐, 3.5‐, 2.4‐, 4.7‐ and 5.5‐folds, respectively, and as development of the seedlings to the four‐ to five‐leaf stage up‐regulation was further increased to 11.7‐, 8.9‐, 9.6‐, 16.2‐, 222.6‐ and 22‐fold higher, respectively, than in WT (Figure 2e). Thus, we speculate that the defence responses are triggered in *spl35* and that the uncontrolled continuation of the responses leads to the development of mimic lesions and enhanced disease resistance.

Shi *et al*. (2013) showed that expression of *LOC_Os03g10750* (*SPL35*) was suggested a role in abiotic stress. In order to evaluate response of *SPL35* to other abiotic stresses, we examined expression levels of *SPL35* in wild‐type Kinmaze seedlings at different time intervals after treatments with mannitol, H_2_O_2_, NaCl, 1‐Amino‐1‐cyclopropanecarboxylic acid (ACC) and abscisic acid (ABA), and showed that *SPL35* was significantly induced by these stresses at 4 h post treatment (Figure S5). Based on these aforementioned results, we speculate that *SPL35* may be involved in various signalling or regulatory pathways associated with biotic and abiotic stress.

### Identification of the *spl35* locus

**Figure 3:**
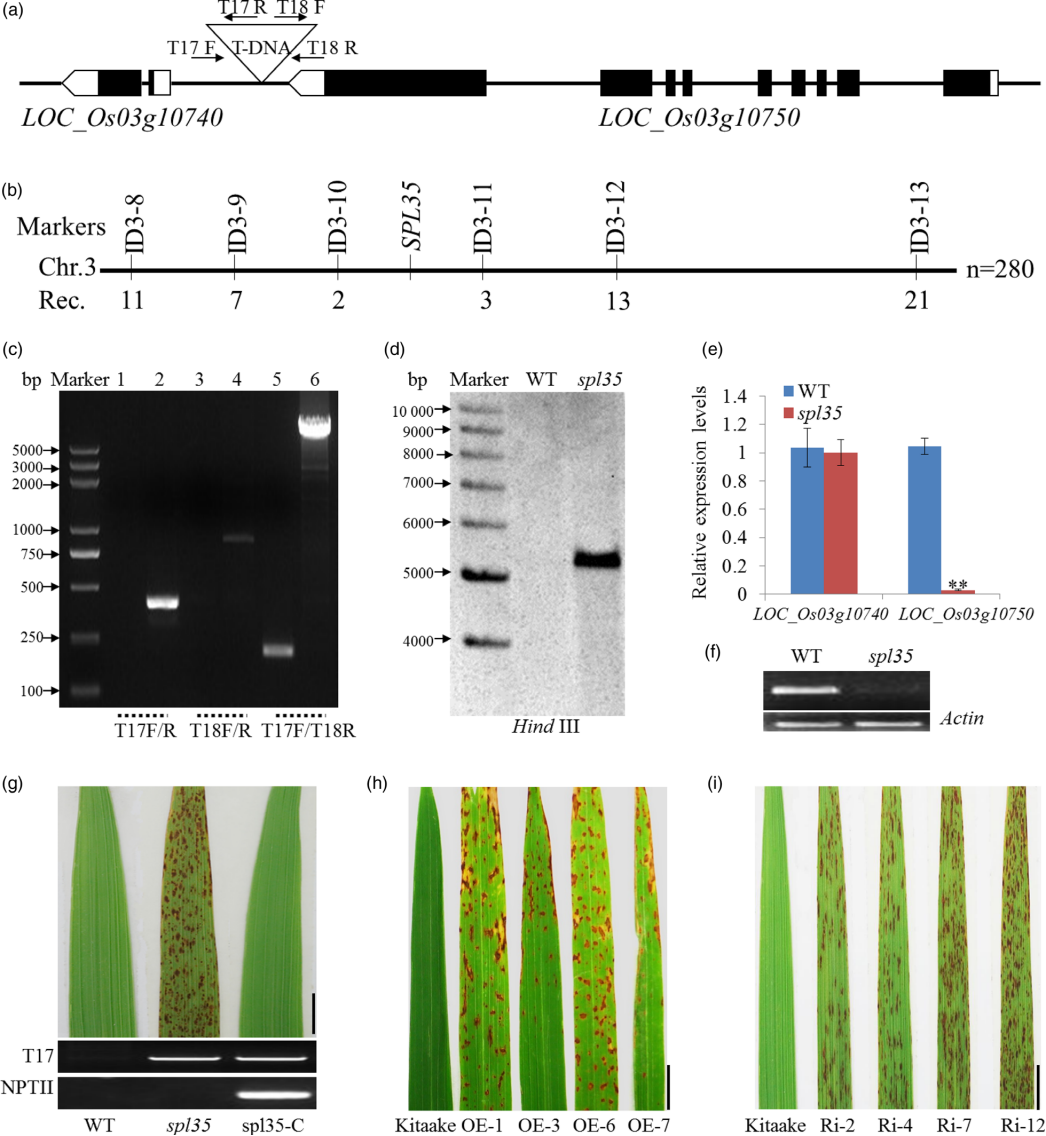
Cloning and molecular analysis of the *SPL35* gene. (a) T‐DNA insertion site of the *spl35* mutant. The triangle indicates the T‐DNA insertion site in the *spl35* mutant. Black boxes indicate exons, lines indicate introns, and white boxes indicate the untranslated regions. (b) Physical map of the *SPL35* locus on the short arm of chromosome 3. Numbers below the map indicate the number of recombinants between markers. (c) Flanking sequence was confirmed by PCR using the primers (T17F/R, T18F/R and T17F/T18R) shown in (a). Lanes 1, 3 and 5 represent WT; Lanes 2, 4 and 6 represent *spl35*; M, DL2000 marker. (d) Copy number determination of T‐DNA insertion by Southern blotting. A fragment of the hygromycin phosphotransferase gene was used as probe. The genomic DNA was digested by *Hind* III. (e) Expression of two genes within the vicinity of the T‐DNA insertion site by qRT‐PCR analysis. Asterisks indicate significant differences between WT and *spl35* plants (**P < 0.01; Student's *t* test). Data are means ± SD of three independent biological replicates. (f) Expression analysis of *LOC_Os03g10750* in WT and *spl35* plants by qRT‐PCR. Rice actin gene was used as an internal control. (g) Phenotypes of WT,* spl35* and complemented *spl35* (*spl35‐C*) plants at the 15^th^ day after flowering in field conditions. *Scale bars*, 5.0 cm. (h, i) Lesion mimics on the flag leaves of *LOC_Os03g10750*‐OE and ‐Ri T_1_ lines in the cv. Kitaake background. Data are means ± SD of three independent biological replicates. *Scale bars*, 1.0 cm.

Since *spl35* was isolated from a T‐DNA population, we first determined if the lesion mimic phenotype was associated with the presence of the T‐DNA insertion using the hygromycin B marker Hyr‐F/R and number of insertions in this line by Southern blotting. Our analysis indicated that the mutation was recessive, caused by a single T‐DNA insertion (Table S4; Figure 3d). Next, we employed the TAIL‐PCR method and cloned a 750 bp sequence flanking the T‐DNA. BLAST analysis of this sequence (http://blast.ncbi.nlm.nih.gov/Blast.cgi) anchored the insertion to a site 948 bp upstream of the translation initiation site of *LOC_Os03g10740* and 458 bp downstream of the translation termination site of *LOC_Os03g10750* (Figure 3a,c). Furthermore, using 280 F_2_ lesion mimic individuals from the cross of *spl35* with an *indica* cv. 93‐11, we were able to delimit the mutation locus to a 760 kb region flanked by markers ID3‐10 and ID3‐11 (location: 4 894 747–5 654 369 bp; Figure 3b). This region spans the T‐DNA site, thus supporting the TAIL‐PCR result.

We performed qRT‐PCR and semi‐quantitative RT‐PCR to understand if the T‐DNA insertion caused any change in expression level of *LOC_Os03g10750* and *LOC_Os03g10740* and found significant down‐regulation of *LOC_Os03g10750* (Figure 3e–f). To verify if the down‐regulation of *LOC_Os03g10750* was the cause of the mutant phenotype, we introduced the constructs *p2300C‐10750* containing a genomic sequence of *LOC_Os03g10750*, and *p2300C‐10740* containing a genomic sequence of *LOC_Os03g10740* as a comparison, into *spl35*, and obtained 22 and 15 independent transgenic plants, respectively. Complementation was found in all the *p2300C‐10750*, but not *p2300C‐10740* plants, suggesting that *LOC_Os03g10750* was the candidate gene for *SPL35* (Figure 3g; Figure S6).

### *SPL35* encodes a CUE domain‐containing protein

Sequence comparison between genomic DNA and cDNA showed that *SPL35* contains nine exons and eight introns (Figure 3a). The coding sequence of *SPL35* consists of 2784 nucleotides encoding a 927 amino acid protein with an estimated molecular weight of 101.95 kD, and a pI of 6.65 (Figure S7a). SPL35 is an unknown protein containing a single CUE domain of 43 amino acids that shares 46.5% similarity to the Cue1p‐CUE domain first identified in yeast (Biederer *et al*., 1997; Figure S7a,b). The SPL35 protein also contains conserved Phe‐Pro (FP) and Ile‐Leu (IL) motifs that are crucial for efficient ubiquitin binding, suggesting that it may function in ubiquitin binding. The SPL35 CUE domain is also similar to that of the CUEDC proteins Cue2, Tollip and Vps9 which are involved in the trafficking and ubiquitination pathways (Shih *et al*., 2003; S7b). A Gramene database (http://ensembl.gramene.org/) search showed that there were five other putative CUEDC proteins in rice, LOC_Os02g09180, LOC_Os06g43550, LOC_Os10g16440, LOC_Os06g49200 and LOC_Os07g30100, but they are different from SPL35 in amino acid sequence (Figure S7b). Phylogenetic analysis revealed that SPL35 has the higher similarity to orthologs from monocot plants species such as *Brachypodium distachyon* (78.9%), *Hordeum vulgare* (50.2%) *Sorghum bicolor* (77.4%) and *Zea mays* (62.8%), but much lower similarity to orthologs in dicot species (29.2%–59.8%) and animals (28.8%–35.3%; Figure S8).

### Both overexpression and knockdown of *SPL35* cause the lesion mimic phenotype

To examine the expression of *SPL35* is tightly regulated for its normal function, we transformed an overexpression vector (*p35S:SPL35‐OE*) and an RNA interference vector (*pUbi:SPL35‐RNAi*) into cv. Kitaake, and produced 16 and 21 independent plants, respectively. Overexpression in the *p35S:SPL35‐OE* plants and down‐regulation in the *pUbi:SPL35‐RNAi* plants were confirmed by qRT‐PCR (Figure S9). As expected, down‐regulation of *SPL35* caused the lesion mimic phenotype, but to our surprise, overexpression of *SPL35* also lead to a phenotype similar to *spl35* such as decreased tiller number and plant height in addition to the mimic lesions (Figure 3h,i;Figure  S10). Those findings suggest that expression of *SPL35* must be tightly regulated to ensure its function and that manipulating expression level of *SPL35* may be a potential strategy for improving disease resistance in plant breeding.

In addition, we did deletion analysis to SPL35 by overexpressing its N‐terminal (1‐568) containing the CUE domain (*p35S:SPL35* ^*1‐568*^ *‐OE*) and its C‐terminal (569‐927) (*p35S:SPL35* ^*569‐927*^ *‐OE*) in cv. Kitaake. Sixteen of the 19 *p35S:SPL35* ^*1‐568*^ *‐OE* plants, but none of the 15 *p35S:SPL35* ^*569‐927*^ *‐OE* plants, showed a phenotype similar to *spl35* (Figure S11). This result implies an essential role of the CUE domain for functioning of SPL35.

### Expression pattern of *SPL35*

**Figure 4:**
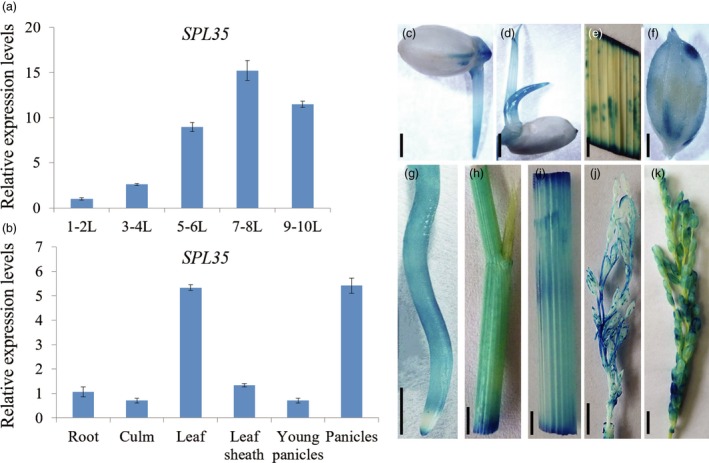
Expression pattern of *SPL35* in the wild‐type Kinmaze. (a) Expression levels of *SPL35* in leaf blades at different developmental stages. L, leaf blade. Each bar is the mean ± SD of three independent biological replicates. (b) Expression levels of *SPL35* in various tissues. Data are means ± SD of three independent biological replicates. (c–k) Gus staining of *SPL35*pro::GUS transgenic plants. Seed (c), young leaf (d), leaf blade (e), hull (f), root (g), leaf sheath (h), culm (i), 8–10 cm young panicle (j) and mature panicle (k). *Scale bars*, 1.0 mm.

We investigated the expression pattern of *SPL35* at different development stages and in different organs by qRT‐PCR. The transcription level of *SPL35* in leaf blades was low at the one‐ to two‐leaf stage, reached a peak at the seven‐ to eight‐leaf stage, and then decreased at the nine‐ to ten‐leaf stage (Figure 4a), indicating developmental regulation of *SPL35*. *SPL35* was expressed in all organs examined, with the strongest expression in leaf blades and panicles (Figure 4b). We also generated transgenic plants carrying the *pSPL35:GUS* reporter construct. β‐glucuronidase (GUS) staining showed activity of the *SPL35* promoter in all tissues examined (Figure 4c–k). These results indicate that *SPL35* is ubiquitously expressed in all organs and to certain extent under developmental regulation.

### Subcellular localization of the SPL35 protein

**Figure 5:**
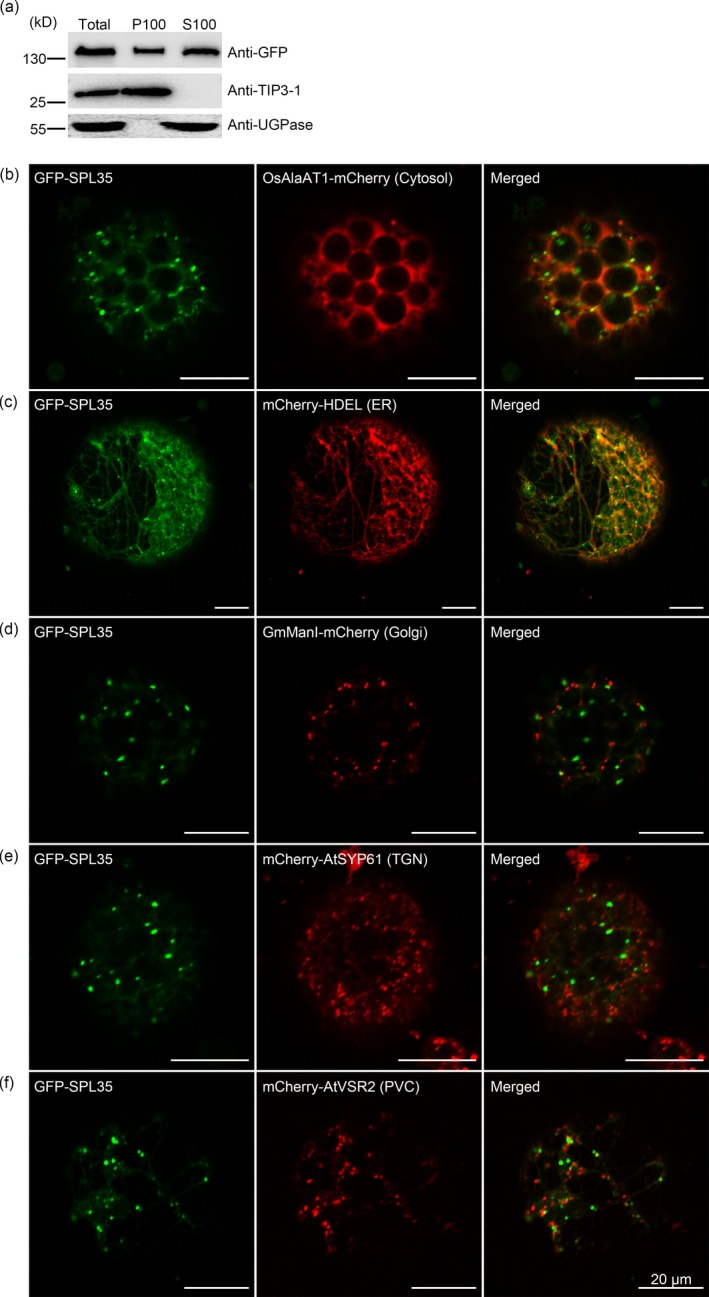
Subcellular localization of SPL35 in *Nicotiana benthamiana* leaf epidermal cells. (a) SPL35 may be a peripherally membrane‐associated protein. Total extract from rice protoplasts was ultracentrifuged at 100 000 ***g*** for 1 h to obtain the pellets (P100) and supernatants (S100) fractions, followed by immunoblot analysis with anti‐GFP and antibodies as indicated. (b–f) Co‐expression of GFP‐SPL35 and OsAlaAT1‐mCherry (Cytosol marker), mCherry‐HDEL (ER marker), GmManI‐mCherry (Golgi marker), mCherry‐AtSYP61 (TGN marker) and mCherry‐AtVSR2 (PVC marker) in *Nicotiana benthamiana* epidermal cell‐derived protoplasts. *Scale bars*, 20 μm.

SPL35 has no predicted transmembrane domain except the only CUE domain (http://smart.embl-heidelberg.de/smart). Transient expression of *p35S:GFP‐SPL35* in rice protoplasts and immunoblot analysis showed that the GFP‐SPL35 protein was found in both soluble and microsomal fractions, as detected by antibodies specific to the vacuolar membrane marker TIP3‐1 and cytosol marker UGPase (Figure 5a), suggesting that SPL35 could be a protein that locates at multiple places rather than an integral membrane protein.

To visualize the intracellular localization of SPL35, we transiently expressed *p35S:GFP‐SPL35* in *Nicotiana benthamiana* (*N. benthamiana*) epidermal cells. The GFP‐SPL35 fusion protein was predominantly localized in cytosol, ER and punctate compartment(s), as indicated by overlap between GFP signals and red signals from the cytosol marker (OsAlaAT1‐mCherry, Zhong *et al*., 2019) and the ER marker (mCherry‐HDEL, Nelson *et al*., 2007; Figure 5b,c). To further understand the nature of the punctate compartment, we coexpressed GFP‐SPL35 with other fluorescent markers, such as for Golgi apparatus (GmManI‐mCherry, Tse *et al*., 2004), TGN (mCherry‐AtSYP61, Lam *et al*., 2007) or PVC (mCherry‐VSR2, Miao *et al*., 2006). As a result, the GFP‐SPL35 punctate structures merged with none of these organelles (Figure 5d–f), indicating that the punctate compartment is not Golgi apparatus, TGN or PVC.

### SPL35 interacts with monoubiquitin through the CUE domain

**Figure 6:**
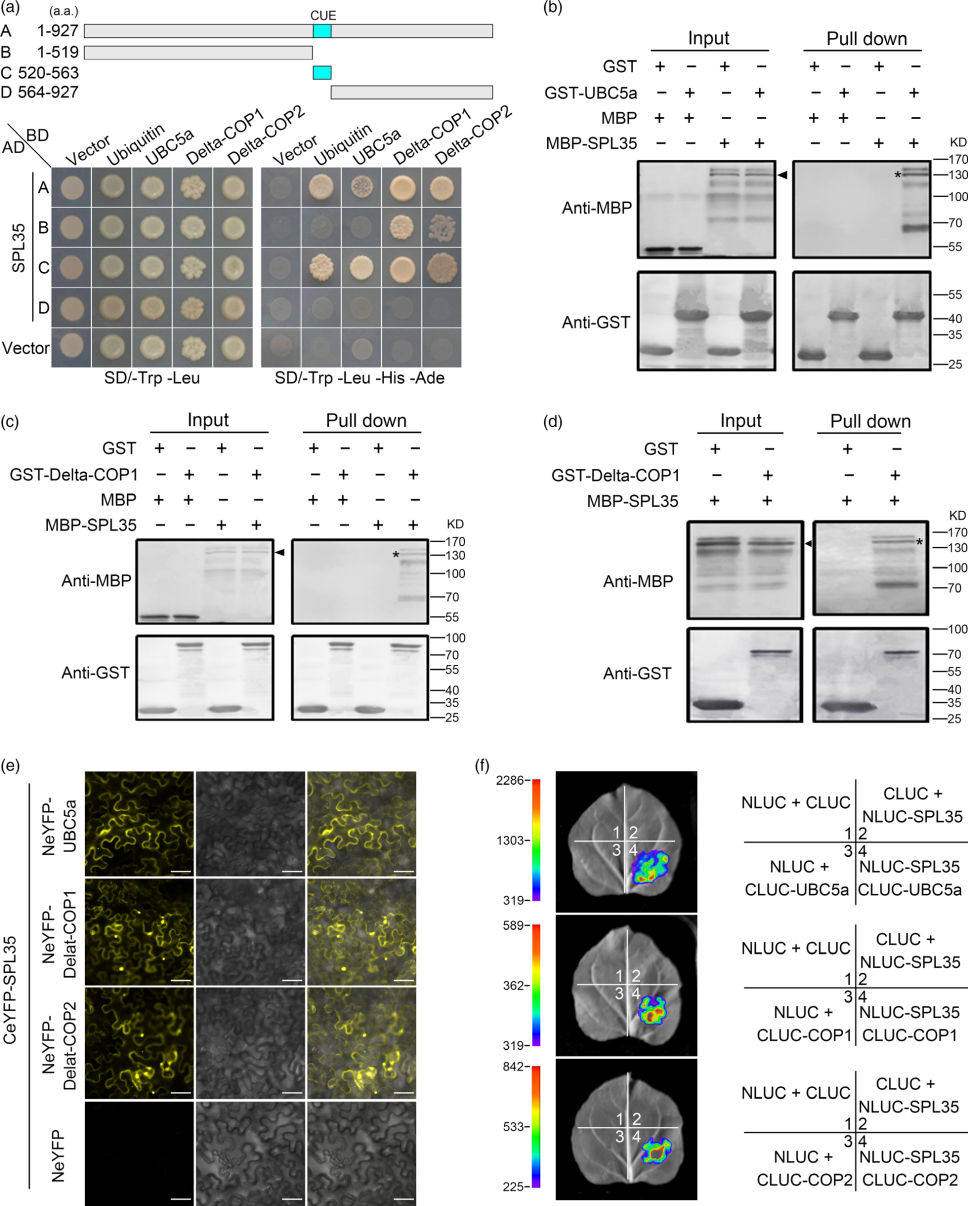
Interaction of SPL35 with OsUBC5a, Delta‐COPI and Delta‐COP2. (a) Yeast two‐hybrid assays for interaction of SPL35 with Ubiquitin, OsUBC5a, Delta‐COP1 or Delta‐COP2. The full‐length and different fragments of SPL35 were inserted to the prey (AD) vector pGADT7, and the ubiquitin, OsUBC5a, Delta‐COP1 and Delta‐COP2 were inserted into vector pGBKT7 as baits. Yeast strains were cultured on the SD/–Trp‐Leu and SD/–Trp‐Leu‐His‐Ade selection medium with 3 mm 3‐amino‐1,2,4‐triazole. (b–d) Pull‐down assays for the interactions of SPL35 with OsUBC5a, Delta‐COP1 and Delta‐COP2 *in vitro* respectively. GST (26.30 kD), GST‐OsUBC5a (42.87 kD), GST‐ Delta‐COP *1* (84.07 kD), GST‐ Delta‐COP2 (84.07 kD), MBP (42.5 kD) and MBP‐SPL35 (143.25 kD) were expressed in bacteria. Pull‐down was performed using GST binding resin. Proteins were detected with antibodies as indicated. Similar results were obtained in three independent experiments. The asterisk shows the band pulled down, and the arrow shows the immune‐blotting band of MBP‐SPL35 by the anti‐MBP monoclonal antibody. (e) Interactions between SPL35 and OsUBC5a, Delta‐COP1 and Delta‐COP2 shown by BiFC assays in *Nicotiana benthamiana* leaf epidermal cells. BiFC fluorescence is indicated by the eYFP signal. The signal of eYFP was not detected in the corresponding negative controls (fourth panels). eYFP, enhanced yellow fluorescent protein fluorescence; DIC, differential interference contrast. *Scale bars*, 10 μm. (f) firefly LUC complementation imaging (LCI) assay detecting the interaction between SPL35 and OsUBC5a, Delta‐COP1 and Delta‐COP2. The coloured scale bar indicates the luminescence intensity. NLUC indicates the N terminus of LUC, while CLUC indicates the C terminus of LUC.

Monoubiquitination is an important cellular regulatory signal that adds single ubiquitin molecules to a target protein, and regulates vesicle budding and histone modification among other functions (Hicke, 2001). Previous studies showed that CUE domains recognize monoubiquitin signals and directly interact with monoubiquitin (Shih *et al*., 2003). To verify whether the SPL35 protein interacts with ubiquitin, we generated four SPL35 prey vectors containing full‐length or truncated fragments of SPL35, and used the entire coding region of ubiquitin as bait for yeast two‐hybrid assays. Both the full‐length and the CUE domain of SPL35 exhibited strong interaction, but the C‐ or N‐terminal region lacking of the CUE domain showed no interaction with the ubiquitin LOC_Os09g39500 (Figure 6a). This indicates that the CUE domain is required for the interaction of SPL35 with ubiquitin in yeast cells, and thus SPL35 could be involved in monoubiquitination.

### SPL35 directly interacts with OsUBC5a, Delta‐COP1 and Delta‐COP2

We used the full‐length SPL35 in the pGBKT7 vector as bait to screen a rice cDNA library in yeast to look for proteins interacting with SPL35, and identified three genes, *LOC_Os01g46926*,* LOC_Os05g24594* and *LOC_Os05g24601*. *LOC_Os01g46926* encodes a rice ubiquitin conjugation enzyme, *OsUBC5a* (Takai *et al*., 2002). *LOC_Os05g24594* and *LOC_Os05g24601* encode rice clathrin‐associated proteins Delta‐COP1 (Q0DJA0) and Delta‐COP2 (Q0DJ99) respectively (http://www.uniprot.org/uniprot/).

We generated three bait vectors each containing full‐length CDS of *OsUBC5a*,* Delta‐COP1* or *Delta‐COP2*, to verify their interaction with SPL35. OsUBC5a interacted with both full‐length and the CUE domain‐containing truncation of SPL35; Delta‐COP1 and Delta‐COP2 interacted with the full‐length, N‐terminal region and CUE domain‐containing truncations of SPL35 (Figure 6a). To determine whether they interact directly to each other, we performed glutathione S‐transferase (GST) pull‐down assay *in vitro*. Purified recombinant proteins GST‐OsUBC5a, GST‐Delta‐COP1 and GST‐Delta‐COP2 were separately immobilized to glutathione‐sepharose beads. The SPL35 protein tagged with maltose‐binding protein (MBP) was purified and incubated with each bead preparation *in vitr*o. MBP and GST alone were used as negative controls. Immunoblot assays showed that a 128.24 kD band was recognized by an antibody against MBP, and MBP‐SPL35 was pulled down (Figure 6b–d), indicating that SPL35 directly and independently interacted with OsUBC5a, Delta‐COP1 or Delta‐COP2 *in vitro*.

To further verify the interaction between SPL35 and OsUBC5a, Delta‐COP1 or Delta‐COP2, we performed subcellular localization of OsUBC5a, Delta‐COP1 and Delta‐COP2, and BiFC assays. We firstly fused the coding regions of OsUBC5a, Delta‐COP1 or Delta‐COP2 to the C terminus of green fluorescent protein (GFP) to produce the GFP‐OsUBC5a, GFP‐Delta‐COP1 or GFP‐Delta‐COP2 fusion construct under control of the CaMV 35S promoter. We observed fluorescence signals of GFP‐OsUBC5a, GFP‐Delta‐COP1 and GFP‐Delta‐COP2 with similar localization of the SPL35 protein (Figure S12). BiFC assays showed that SPL35 interacted with OsUBC5a, Delta‐COP1 and Delta‐COP2 in *N. benthamiana* leaf cells (Figure 6e). Furthermore, the firefly LUC complementation imaging (LCI) assay confirmed the *in vivo* interaction of SPL35 and three interactors in leaf epidermal cells of *N. benthamiana* (Figure 6f). These results suggest that SPL35 directly interacts with OsUBC5a, Delta‐COP1 and Delta‐COP2 *in vivo*, and may be involved in the protein ubiquitin and transport pathways.

**Figure 7:**
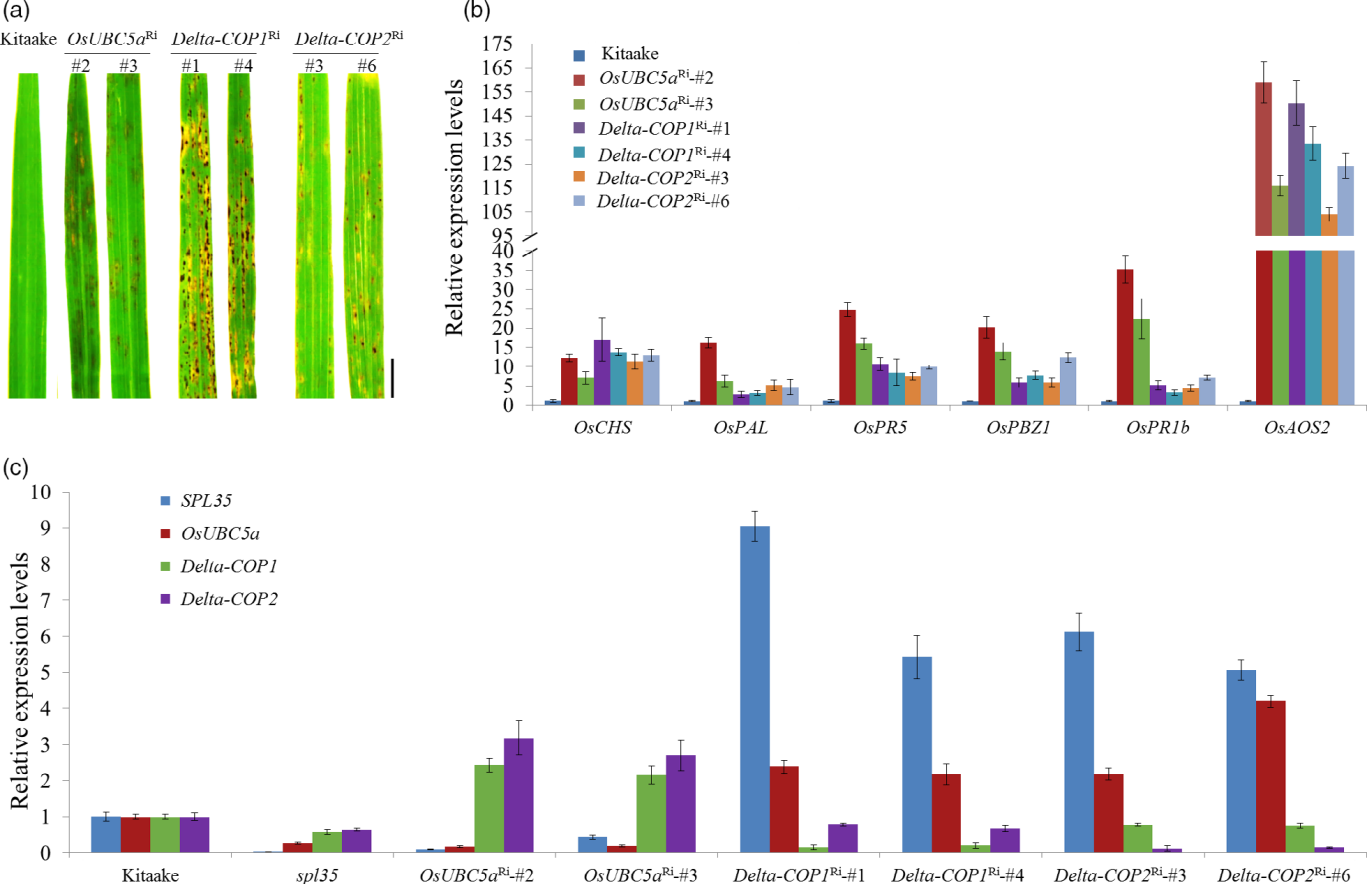
Expression levels of three *SPL35*‐interacting genes and six defence‐/ pathogenesis‐related genes in wild‐type, *spl35* or RNAi transgenic T_1_ plants showing lesion mimic phenotypes. (a) Lesion mimic phenotypes of the flag leaves of 60‐day‐old plants of the receptor genotype (cv. Kitaake) and RNAi (*OsUBC5a* ^Ri^, *Delta‐COP1* ^Ri^ and *Delta‐COP2* ^Ri^) T_1_ transformants in the greenhouse. *Scale bars*, 1.0 cm. (b) qRT‐PCR analysis of defence‐/ pathogenesis‐related genes in the receptor genotype (cv. Kitaake) and RNAi (*OsUBC5a* ^Ri^, *Delta‐COP1* ^Ri^ and *Delta‐COP2* ^Ri^) T_1_ transformants respectively. Similar results were obtained in three independent biological replicates. (c) Expression levels of *SPL35*,* OsUBC5a*,* Delta‐COP1* and *Delta‐COP2* genes in Kitaake, *spl35* and RNAi transgenic T_1_ plants. Data are means ± SD of three independent biological replicates. *OsUBC5a* ^Ri^‐#2 and *OsUBC5*a^Ri^‐#3 are *OsUBC5a* RNAi transgenic plants. *Delta‐COP1* ^Ri^‐#1 and *Delta‐COP1* ^Ri^‐#4 are *Delta‐COP1* RNAi transgenic plants. *Delta‐COP2* ^Ri^‐#3 and *Delta‐COP2* ^Ri^‐#6 are *Delta‐COP2* RNAi transgenic plants.

To understand the functions of *OsUBC5a*,* Delta‐COP1* and *Delta‐COP2*, we transformed the corresponding RNAi constructs (Table S1) into cv. Kitaake to knock down their expression. We obtained 17, 12 and 15 independent RNAi plants for *OsUBC5a*,* Delta‐COP1* and *Delta‐COP2* respectively. All the RNAi plants displayed cell death phenotypes resembling that of s*pl35* (Figure 7a), suggesting that these SPL35‐interacting proteins function in regulating cell death in rice.

To probe into the mechanisms that caused the cell death phenotypes in the RNAi plants of *OsUBC5a*,* Delta‐COP1* and *Delta‐COP2*, we first examined the expression of these genes together with *SPL35* in WT during *M. oryzae* and *Xoo* infections and in their respective knockdown mutants. The expression patterns of these genes were not obviously affected by the pathogen infections (S13). Then, we examined the expression of six DR/PR genes, *OsCHS*,* OsPAL*,* OsPR5*,* OsPBZ1*,* OsPR1b* and *OsAOS2*, in the *OsUBC5a* ^Ri^; *Delta‐COP1* ^Ri^ and *Delta‐COP2* ^Ri^ plants. The expression levels of these DR/PR genes were all obviously up‐regulated in the RNAi plants, relative to WT (cv. Kitaake; Figure 7b), suggesting an activation of the defence response. The mRNA levels of *OsUBC5a*,* Delta‐COP1* and *Delta‐COP2* were dramatically decreased in *spl35* relative to WT, and the expression levels of both *SPL35* and *OsUBC5a* were significantly down‐regulated, whereas those of both *Delta‐COP1* and *Delta‐COP2* were significantly up‐regulated in the *OsUBC5a* ^Ri^ plants; however, the expression levels of both *SPL35* and *OsUBC5a* were significantly up‐regulated in both *Delta‐COP1* ^Ri^ and *Delta‐COP2* ^Ri^ plants (Figure 7c). These results reflected the complex mutual influences between *SPL35*, O*sUBC5a*,* Delta‐COP1* and *Delta‐COP2*, and suggested that the imbalance in the expression of *SPL35* might be a key reason for the cell death phenotypes in the *OsUBC5a*,* Delta‐COP1* and *Delta‐COP2* RNAi plants.

## Discussion

### *SPL35* encodes a novel CUE domain‐containing protein in plants

LMMs are ideal materials for investigating HR‐mediated cell death and defence responses in plants. In the present study, we identified a LMM *spl35* in a T‐DNA insertion mutant population of rice cv. Kinmaze. This mutant exhibited spontaneous HR‐like lesions on leaves from the three‐leaf stage seedlings through the ripening stage, accompanied by decreased plant height, fewer panicles, lower grain number and weight, and reduced spikelet fertility (Figure 1; Table S2). *SPL35* was identified as *LOC_Os03g10750*, which encodes a CUEDC protein. Functional complementation with wild‐type *LOC_Os03g10750* rescued the *spl35* phenotype (Figure S6). It is worth noting that both overexpression and down‐regulation of *SPL35* resulted in HR‐like cell death and other features typical of *spl35* (Figure 3h,i; Figures S9 and S10). This indicates that *SPL35* is an important gene, which must be properly regulated to maintain normal plant growth and development.

Five other rice genes are predicted to encode putative CUEDC proteins (Figure S7b). SPL35 has 3.3%–19.8% similarities with those CUEDC proteins; and the CUE domain of SPL35 also differs from those of the other CUEDC proteins (Figure S7b). There are a number of orthologs of *SPL35* in other monocot and dicot plant species (Figure S8), but none of them has been functionally characterized. The duplicated pair of *Arabidopsis* RING‐finger E3 ligases, RIN2 and RIN3, are the only reported CUEDC proteins in plants, but a role of the CUE domains for functioning of RIN2 and RIN3 remains elusive (Kawasaki *et al*., 2005). The present study demonstrates that the CUE domain of SPL35 is required for functioning of SPL35. We also showed that SPL35 directly interacts with the ubiquitin, OsUBC5a, Delta‐COP1 and Delta‐COP2; its dysfunction can activate cell death and defence responses in rice, and significantly enhances resistance to different diseases. To our knowledge, this is the first report that a CUEDC protein is involved in plant immunity and defence response pathways.

### *SPL35* regulates cell death and defence responses

HR‐mediated cell death that commonly occurs at invasion sites to restrict further invasion or proliferation of pathogens, is a major hallmark of defence responses in plants (Wang *et al*., 2017). Identification of LMMs and characterization of the corresponding genes have expanded our understanding of cell death and defence responses in plants. Some CUE domain‐containing proteins were reported to be involved in apoptosis in yeast and mammalian species. For example, CUEDC2 suppresses glioma tumorigenicity by inhibiting the activation of STAT3 and NF‐κB signalling pathway (Li *et al*., 2017), and gp78 acts as a regulator of normal liver homeostasis and a tumour suppressor in human liver (Zhang *et al*., 2015). In order to determine whether SPL35 controls cell death in rice, we performed expression analyses of two histochemical markers using two staining methods, DAB staining and trypan blue staining (Wang *et al*., 2017), and measurement of H_2_O_2_ contents in *spl35* and WT plants respectively. The results provided evidence that SPL35 controls cell death in plants (Figure S2). Considering that *spl35* is characterized by spontaneous cell death in leaves, we conclude that SPL35 protein plays an important role in preventing cell death in rice.

Previous studies suggested that the development of symptoms in LMMs may activate defence response genes, and contribute to enhanced resistance to pathogens (Wang *et al*., 2015a,2015b, 2017). The present study showed that six defence‐related or pathogenesis‐related genes were rapidly activated (Figure 2e), and resistance to multiple *M. oryzae* and *Xoo* isolates was significantly enhanced as HR‐like lesions developed in *spl35* plants (Figure 2a–d; Figure S4), indicating that the induced resistance was broad‐spectrum and perhaps nonspecific. Wang *et al*. (2017) showed that three PR genes, *PR1a*,* PBZ1* and *PO‐C1*, were significantly up‐regulated in *spl33* plants during the development of lesion mimics. A similar result was obtained for *spl35* plants, and up‐regulated expression of six DR/PR genes (Figure 2e) occurred regardless of the presence of actual lesion mimics, although the expression levels increased with development after the two‐ to three‐leaf stage. It is worth mentioning that with the up‐regulated expression of these DR/PR genes, resistance to the *M. oryzae* isolates were coincidentally increased from moderate to high levels in *spl35* plants (Figure S4; Table S3). Therefore, we conclude that *SPl35* may regulate defence responses correlated with mimic lesion development in *spl35*.

### *SPL35* is involved in ubiquitination and vesicular trafficking pathways

Previous studies revealed that the CUE domain has dual roles in mono‐ and poly‐ubiquitination, responsible for protein trafficking and ER‐associated degradation of misfolded proteins in yeasts and mammalian species (Kostova *et al*., 2009; Man and Zhang, 2011). The present transient expression and two‐hybrid assays showed that SPL35 is predominantly localized to the cytosol and the ER (Figure 5b,c), and can directly interact with ubiquitin (Figure 6a). We identified and validated interaction of SPL35 with the OsUBC5a protein, which belongs to the Ubc4/5 subfamily and functions as an E2 enzyme in rice (Takai *et al*., 2002), by four types of assays, namely yeast two‐hybrid, GST pull‐down, BiFC and LCI (Figure 6). OsUBC5a was co‐localized with SPL35, and interacted with both full‐length and the CUE domain truncation of SPL35 *in vitro* and *in vivo* (Figure 6a,b,e,f). Based on this, we concluded that SPL35 interacts with OsUBC5a through its CUE domain. OsUBC5a catalyses auto‐ubiquitination of EL5, a RING‐type E3 ligase, and regulates cell death in root development (Koiwai *et al*., 2007; Takai *et al*., 2002). OsUBC5a also interacted with SPL11, a U‐box type E3 ligase, and regulated plant PCD and defence responses (Bae and Kim, 2013, 2014). Even though further detailed investigation is needed to determine how SPL35 functions in ubiquitination in rice, it is clear that the CUE domain of SPL35 interacts with ubiquitin and OsUBC5a, thus suggesting that *SPL35* could participate in the ubiquitination pathway.

Proteins are synthesized by ribosomes in the rough ER, and transported to the Golgi apparatus and TGN, and then to their final destination compartment by carrier vesicles. Three types of coated vesicles have been identified, including coat protein complex I (COPI)‐, COPII‐ and clathrin‐coated vesicles and their adaptor complexes that are important for sorting and directional transport of proteins in the endocytic and secretory pathways (McMahon and Mills, 2004). In yeast, COPI‐coated vesicles seem to mediate anteretrograde transport within the Golgi apparatus, and to retrieve cargos from the Golgi back to the ER (retrograde direction; Béthune *et al*., 2006). The COPI coat complex consists of at least seven subunits, among which the subunit delta is a stoichiometric component and is essential for eukaryotic cell viability (Faulstich *et al*., 1996). In plants, COPI‐coated vesicles were identified *in situ* and isolated. They were localized to microvesicles surrounding or budding from the Golgi apparatus, and involved in retrograde transport from the Golgi apparatus to ER (Pimpl *et al*., 2000). To date, there have been no reports on functions of the COP complexes in plant cell death. We searched a total of four coatomer subunit delta genes, *Delta‐COP1* (*LOC_Os05g24594*), *Delta‐COP2* (*LOC_Os05g24601*), *Delta‐COP3* (*LOC_Os01g61710*) and *Delta‐COP4* (*LOC_Os08g28080*), in the rice genome (Figure S14; http://www.uniprot.org/uniprot). In this study, we identified and validated SPL35‐interacting proteins Delta‐COP1 and Delta‐COP2 by yeast two‐hybrid, GST pull‐down, BiFC and LCI assays (Figure 6), and found that knockdowns of *Delta‐COP1* or *Delta‐COP2* resulted in lesion mimic phenotypes (Figure 7a), providing evidence that the COPI protein complex is involved in the regulation of HR‐like cell death in rice. Thus, we speculate that SPL35 directly regulates Delta‐COP1 and Delta‐COP2 by protein–protein interaction and participates in COPI‐mediated protein transport pathways. The facts that overexpression and knockdown of *SPL35*, and knockdowns of *OsUBC5a*,* Delta‐COP1* and *Delta‐COP2* all cause lesion mimic phenotypes (Figure 3h,i; Figure 7a; Figure S10), suggest that imbalance in the expression of SPL35 might inhibit proper complex formation with its regulatory protein, thereby disrupting precise control of downstream events like ubiquitination or vesicular trafficking.

To comprehensively elucidate the regulatory mechanism of *SPL35* in prevention of cell death, further analyses of SPL35 and its interacting proteins for their functions, interaction mechanisms and roles in ubiqutination and vesicular trafficking need to be undertaken. Understanding the relationships between SPL35‐mediated protein ubiqutination and/or vesicular trafficking and cell death, in connection with defence response will be part of that effort.

## Experimental procedures

### Plant materials and growth conditions

The *spl35* mutant (line T466) was originally identified among a T‐DNA (vector pCUbi1390) insertion population (T_1_ plants) of *japonica* cv. Kinmaze. Among 16 T_1_ plants derived from T466, 13 were normal and 3 displayed spotted leaves. T_3_ homozygous plants were obtained by selfing the mutant plants and used in later studies. For a specific light treatment the middle parts of leaf blades of seedlings grown in a growth chamber (12 h of light at 30°C/12 h darkness at 20°C) were wrapped in aluminium foil to block the light. The T_2_ individuals derived from the T_1_ mutant plants with normal phenotype but heterozygous genotype of the hygronmycin B marker Hyr‐F/R (Table S1) along with an F_2_ population from a cross between *spl35* and *indica* cv. 93‐11 were used for genetic analysis. All plants, except specified, were grown in a paddy field at the Changping Experimental Station of Institute of Crop Science during April to October, the normal rice‐growing season.

### Transmission electron microscopy

Transverse sections of leaves from plants at 60 DAS grown under the field conditions were used for transmission electron microscopy observation as described previously (Wang *et al*., 2017).

### Pigment and H_2_O_2_ measurements

Fresh flag leaves of *spl35* and WT at 10 DAF were used to measure chlorophyll and carotenoids contents as described by Arnon (1949). H_2_O_2_ contents in leaves were measured in WT and *spl35* at two‐ to three‐leaf stage (*spl35*‐) and four‐ to five‐leaf stage (*spl35*+) using an H_2_O_2_ assay kit (Beyotime Biotech, Shanghai, China) according to the manufacturer's instructions.

### Histochemical assay

Fresh leaves of *spl35* and WT at 10 DAF were used in histochemical assays. Trypan blue staining for cell death, DAB staining for H_2_O_2_ accumulation, and GUS staining were performed as described previously (Wang *et al*., 2017).

### Evaluation of resistance to biotic and abiotic stresses

Two *M. oryzae* isolates CH43 and CH680, virulent to WT, were used to assess resistance of the *spl35* plants to blast disease at the tillering stage by the injection‐inoculation method (Lei *et al*., 1996). The *spl35* along with WT plants were grown at the same time and inoculated leaves of similar development stage with these two isolates. In the extended experiment to investigate when *spl35* initiates a resistant response, six *M. oryzae* isolates, CH43, CH680, CH1971, CH1899, FJ07‐18‐1 and GD02‐15‐1, were separately used to infect *spl35* seedlings using a spray‐inoculation method (Li *et al*., 2008) at the two‐ to three‐leaf and four‐ to five‐leaf stages respectively. Blast disease resistance was scored 7 days post inoculation (Mackill and Bonman, 1992).

Four *Xoo* isolates PXO61, PXO71, PXO76 and PXO86, virulent to WT, were used to assess resistance of the *spl35* plants to blight disease at the booting stage by the scissors‐dipping method (Kauffman *et al*., 1973) or at the seedling stage by the infiltration‐inoculation method (Wang *et al*., 2015c). Lesion lengths were measured 2 weeks post inoculation.

To evaluate responses of *spl35* to abiotic stresses, 14‐day‐old seedlings growing on three layers of Whatman 3 mm filter paper were used for various treatments. The roots of seedlings were dipped in 300 mm Mannitol, 200 mm NaCl or 200 mm H_2_O_2_ solution (Zhang *et al*., 2007). Seedlings were sprayed with 50 μm ACC or 100 μm ABA [dissolved in 0.01% (v/v) ethanol] (Wan *et al*., 2011). RNA was extracted from seedling leaves at 0, 1, 2, 4, 6, 8 and 10 h after treatment.

### TAIL‐PCR and Southern blotting

TAIL‐PCR (Liu *et al*., 1995) was used to isolate regions flanking the T‐DNA insertion with Genome Walking Kit (TaKaRa, Tokyo, Japan). PCR‐amplified fragments were cloned into the pEASY‐Blunt vector (TransGen Biotech, China) and then sequenced (Biomed Biotech, Beijing, China). For Southern blotting, about 10 μg genomic DNA from wild‐type and *spl35* mutant were digested with *Hind*III overnight, and digested products were separated in 1% agarose gels and then transferred onto a Hybond‐N+ membrane (Amersham Pharmacia, Biotech, Buckinghamshire, UK). A 481‐bp fragment of the hygromycin phosphotransferase gene amplified from the pCUbi1390 vector by the primer pair Hyr‐F/R was DIG‐labelled and used as a hybridization probe. Southern blotting was performed with DIG high prime DNA labelling and a detection starter kit II according to the manufacturer's instructions (Roche, Mannheim, Germany). Primers used for TAIL‐PCR and Southern blotting are listed in Table S1.

### Quantitative real‐time PCR (qRT‐PCR) analysis

RNA was extracted from seedling leaves at the four‐leaf stage, flag leaves, leaf sheaths, culms and young panicles at the booting stage. Reverse transcription and qRT‐PCR were performed as described previously (Ma *et al*., 2015). The rice *Ubiquitin* gene (*LOC_Os03g13170*) was used as an internal control (primer pair Ubi), and the 2^−ΔΔCT^ method was used to calculate relative levels of gene expression (Schmittgen and Livak, 2008). Primer pairs designed using GenScript (https://www.genscript.com/ssl-bin/app/primer) are listed in Table S1.

### Vector construction and rice transformation

Two complementation vectors (*p2300C‐10750* and *p2300C‐10740*) were constructed by inserting each of the corresponding genomic DNA fragments from cv. Kinmaze into the *Sac*I and *Sal*I sites of the pCAMBIA2300 vector (http://www.cambia.org/) using In‐Fusion Advantage Cloning Kit (Clontech, Beijing, China). The 8729 bp insertion in *p2300C‐10750* consists of a 2369 bp region upstream of ATG of *LOC*‐*Os03g10750* (*SPL35*), the entire coding region of 5507 bp and an 853 bp region downstream of TGA. The 2414 bp insertion in *p2300C‐*10740 consists of a 1086 bp region upstream of ATG of *LOC*‐*Os03g10740*, the entire gene of 418 and a 910 bp downstream of TGA.

Three overexpression vectors (*p35S:SPL35‐OE*,* p35S:SPL35* ^*1‐568*^ *‐OE* and *p35S:SPL35* ^*569‐927*^ *‐OE*) were constructed by inserting each of the corresponding cDNA fragments from cv. Kinmaze into the *Kpn*I site of the vector pCUbi1390 using In‐Fusion Advantage Cloning Kit (Clontech, Beijing, China). *p35S:SPL35‐OE* contains the full‐length coding DNA sequence (CDS) of *SPL35* (2874 bp); and *p35S:SPL35* ^*1‐568*^ *‐OE* and *p35S:SPL35* ^*569‐927*^ *‐OE* contain a 1704 bp fragment encoding the N‐terminal of *SPL35* (amino acids 1‐568) and a 1080 bp fragment encoding the C‐terminal (amino acids 569‐927) respectively.

Five RNAi vectors (*pUbi:SPL35‐RNAi*,* pUbi:LOC_Os03g10740‐RNAi*,* pUbi:OsUBC5a‐RNAi*,* pUbi:Delta‐COP1‐RNAi*, and *pUbi:Delta‐COP2‐RNAi*) were constructed by inserting each of corresponding cDNA fragments from cv. Kinmaze into the *Sac*I and *SnaB*I sites of binary vector pLH‐FAD2‐1390RNAi using In‐Fusion Advantage Cloning Kit (Clontech, Beijing, China). *pUbi:SPL35‐RNAi* contains a 279 bp cDNA fragment of *SPL35* spanning the bases 995 and 1273; *pUbi:LOC_Os03g10740‐RNAi* contains a 420 bp cDNA fragment of *LOC_Os03g10740* spanning the bases ‐6 and 414; *pUbi:OsUBC5a‐RNAi* contains a 502 bp cDNA fragment of *OsUBC5a* spanning the bases 1 and 502; *pUbi:Delta‐COP1‐RNAi* contains a 301 bp cDNA fragment of *Delta‐COP1* spanning the bases 63 and 363; and *pUbi:Delta‐COP2‐RNAi* contains a 300 bp cDNA fragment of *Delta‐COP2* spanning the bases −115 and 185.

The reporter construct *pSPL35:GUS* was created by amplifying a 2382 bp fragment upstream of the ATG start codon of *SPL35* from cv. Kinmaze, and cloning it into the pCAMBIA1305 binary vector (*Eco*RI and *Nco*I sites) using In‐Fusion Advantage Cloning Kit (Clontech, Beijing, China).

The primers used for building above constructs are listed in Table S1. All the constructs were verified by sequencing and subsequently introduced into *spl35*, Kinmaze or Kitaake by *Agrobacterium tumefaciens*‐mediated transformation as described previously (Nishimura *et al*., 2006).

### Subcellular fractionation and immunodectection

Coding DNA sequence of *SPL35* without the stop codon was amplified and inserted into the *Bgl*II site of the pAN580 vector, generating the fusion construct *pAN35S:GFP‐SPL35*. *pAN35S:GFP‐SPL35* was transformed into callus‐derived rice protoplasts as described previously (Zhou *et al*., 2013). Subcellular fractionation was performed as described previously (Wang *et al*., 2016). Total extract, pellets (P100) and supernatants (S100) were separated by SDS‐PAGE and transferred to PVDF membrane (0.22 μm; Millipore, Shanghai, China), followed by incubation with antibodies against GFP (diluted 1:4000; Roche, Mannheim, Germany), TIP3‐1 (diluted 1:2000; Ren *et al*., 2014) and UGPase (diluted 1:3000; Agrisera, Vännäs, Sweden).

### Transient expression analysis in *N. benthamiana*

The CDS of *SPL35*,* OsUBC5a*,* Delta‐COP1* or *Delta‐COP2* was amplified and inserted into the pCAMBIA1305‐GFP vector (*Bgl*II site) to produce *p35S:GFP‐SPL35*,* p35S:GFP*‐*OsUBC5a*,* p35S:GFP‐Delta‐COP1* or *p35S:GFP‐Delta‐COP2* using In‐Fusion Advantage Cloning Kit (Clontech, Beijing, China) as described previously (Ren *et al*., 2014). Suspension cultures of the *Agrobacterium* strain GV3101 carrying above constructs and organelle markers together with the p19 strain carrying the gene‐silencing suppressor, p19 protein, were infiltrated into 5‐week‐old *N. benthamiana* leaves as described previously (Liu *et al*., 2010). *Nicotiana benthamiana* protoplasts were isolated as described previously (Ren *et al*., 2014). Fluorescence signals were detected using a laser confocal scanning microscopy (ZEISS Microsystems LSM 700, https://www.zeiss.com/microscopy/int/home.html). The primers used for building the above constructs are listed in Table S1.

### Yeast two‐hybrid assay

A rice seedling cDNA library was constructed in yeast using the BD Matchmaker library construction and screening kit (Clontech, Beijing, China). The entire ORF of *SPL35* was cloned into pGBKT7 for screening the cDNA library. *SPL35* CDS fragments were cloned into the pGADT7 vector as preys, and CDSs of *LOC_Os09g39500* (ubiquitin gene), *OsUBC5a*,* Delta‐COP1* and *Delta‐COP2* were inserted into pGBKT7 as baits, respectively. For two‐hybrid assays, the bait and prey vectors were co‐transformed into yeast AH109‐competent cells. Transformed cells were incubated on SD/‐Leu‐Trp‐plates at 30 °C for 2 days, and then transferred to the restrictive medium [SD/‐His‐Ade‐Leu‐Trp‐plates with 3 mm 3‐amino‐1,2,4‐triazole (3‐AT)] and cultured at 30 °C for another 2–4 days. Three repeats were performed. Primers used are listed in Table S1.

### Pull‐down assay

Coding DNA sequence of *SPL35* was inserted into the pMAL‐c2x vector to generate the construct MBP‐SPL35. CDSs of *OsUBC5a*,* Delta‐COP1* and *Delta‐COP2* were cloned into the pGEX4T‐1 vector to generate GST‐OsUBC5a, GST‐Delta‐COP1 and GST‐Delta‐COP2 respectively. The constructs, together with the empty vector, were transformed into *Escherichia coli* BL21 competent cells (TransGen Biotech, Beijing, China) to express protein. Recombinant proteins from *E. coli* lysates were purified using the corresponding resins [amylose resin for MBP purification (New England Biolabs, Beijing, China), and GST‐binding resin for GST purification (Merck, Beijing, China)]. GST or GST fusion proteins coupled to beads were incubated with MBP or MBP‐SPL35. *In vitro* GST pull‐down analysis was performed as described previously (Wang *et al*., 2009). For protein detection, anti‐GST antibody (1:1000), anti‐MBP antibody (1:500) and goat anti‐mouse HRP‐conjugated antibody (1:5000; MBL International, Beijing, China) were used. Three independent repeats were performed. Primers used are listed in Table S1.

### BiFC and LCI assays

For the BiFC assay, CDS of *SPL35* was cloned into the pCCFP‐X (eYFP) vector to construct a CeYFP‐SPL35 fusion. The CDSs of *OsUBC5a*,* Delta‐COP1* and *Delta‐COP2* were cloned into the pNYFP‐X (eYFP) vector to construct NeYFP‐OsUBC5a, NeYFP‐Delta‐COP1 and NeYFP‐Delta‐COP2 fusions respectively. BiFC was performed in *N. benthamiana* leaf epidermis cells as described previously (Liu *et al*., 2010). eYFP signals were observed 48 h after infiltration with a laser confocal scanning microscope (ZEISS Microsystems LSM 700). Primers used are listed in Table S1.

For the LCI assay, CDS of SPL35 was cloned into the NLUC construct to produce the NLUC‐SPL35 fusion, while the CDSs of OsUBC5a, Delta‐COP1 and Delta‐COP2 were separately cloned into the CLUC construct to generate the CLUC‐OsUBC5a, CLUC‐Delta‐COP1 and CLUC‐Delta‐COP2 fusions. LCI assay was performed in *N. benthamiana* leaf epidermis cells. LUC activities were analysed 48 h post‐infiltration using Night‐SHADE LB 985 (Berthold, Bad Wildbad, Germany).

## Conflicts of interest

The authors declare no conflicts of interest.

## Supporting information


**Figure S1** Pigment contents and transmission electron microscopy (TEM) analysis of chloroplasts in wild‐type (WT) and *spl35* mesophyll cells.

**Figure S2** The expression of histochemical markers and measurement of H_2_O_2_ in wild‐type (WT) and *spl35* mutant.

**Figure S3** Expression analysis of three endoplasmic reticulum (ER) chaperone genes in the wild‐type (WT) and *spl35* plants by quantitative real‐time PCR (qRT‐PCR).

**Figure S4** Disease reactions of the wild‐type (WT) and *spl35* mutant to six *Maganaporthe oryzae* isolates.

**Figure S5** Expression of *SPL35* in response to different abiotic treatments.

**Figure S6** Complementation assays of *SPL35* transgenic plants.

**Figure S7** *SPL35* encodes a CUE domain‐containing protein.

**Figure S8** Phylogenetic analysis of SPL35 with other homologues.

**Figure S9** Transcription levels of the *LOC_Os03g10750* gene in different overexpressed (OE) or RNAi (Ri) transgenic lines (T_1_) detected by qRT‐PCR.

**Figure S10** Morphology of *LOC_Os03g10750‐*overexpressed (OE) and ‐RNAi (Ri) transgenic lines (T_1_) in Kitaake background under field conditions. *Scale bars*, 5.0 cm.

**Figure S11** Lesion mimics on the flag leaves of the transgenic lines (T_1_) overexpressing the N‐terminal 1‐568 amino acids of SPL35 in the cv. Kitaake (wild‐type) background OEN‐2, 3 and 5 represent the leaves of pSpl35^1‐568^‐OE transgenic T_1_ lines in cv. Kitaake background.

**Figure S12** Subcellular localization of GFP‐OsUBC5a, GFP‐Delta‐COP1 and GFP‐Delta‐COP2 fusion proteins in *Nicotiana benthamiana* leaf epidermal cells. GFP protein itself is distributed throughout the nucleus and cytoplasm (fourth panels). GFP, green fluorescent protein; DIC, differential interference contrast. *Scale bars*, 10 µm.

**Figure S13** qRT‐PCR analysis of transcript levels of *SPL35*,* OsUBC5a*,* Delta‐COP1* and *Delta‐COP2* during *Maganaporthe oryzae* (*M. oryzae*) and *Xanthomonas oryzae* pv*. oryzae* (*Xoo*) infections.

**Figure S14** The relationship of four coatomer subunit delta proteins in rice by unrooted phylogenetic tree analysis.

**Table S1** Sequences of primers used in this study.

**Table S2** Comparison of agronomic traits between *spl35* mutant and its wild‐type (WT, cv. Kinmaze).

**Table S3** Disease reactions of the *spl35* mutant to six *M. oryzae* isolates.

**Table S4** Genetic analysis of the *spl35* mutant.
