# Supplementary figures and images for: Disruption of gene SPL35, encoding a novel CUE domain‐containing protein, leads to cell death and enhanced disease response in rice
Source: Plant Biotechnol J. 2019 Mar 5;17(8):1679–93. doi: 10.1111/pbi.13093 (PMC6662554; doi:10.1111/pbi.13093)

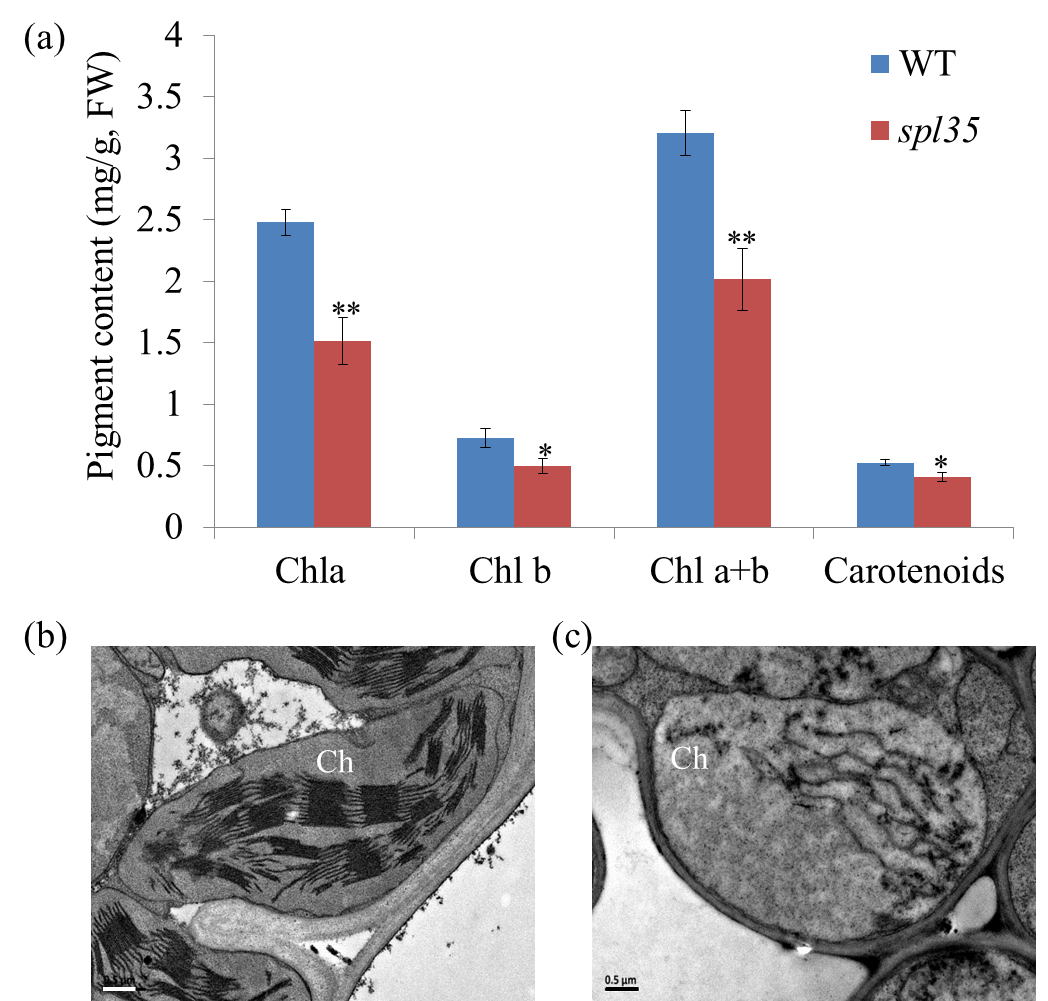

Supplement: Supplementary file 1 — Figure S1 Pigment contents and transmission electron microscopy (TEM) analysis of chloroplasts in wild‐type (WT) and spl35 mesophyll cells. [file PBI-17-1679-s015.tif]

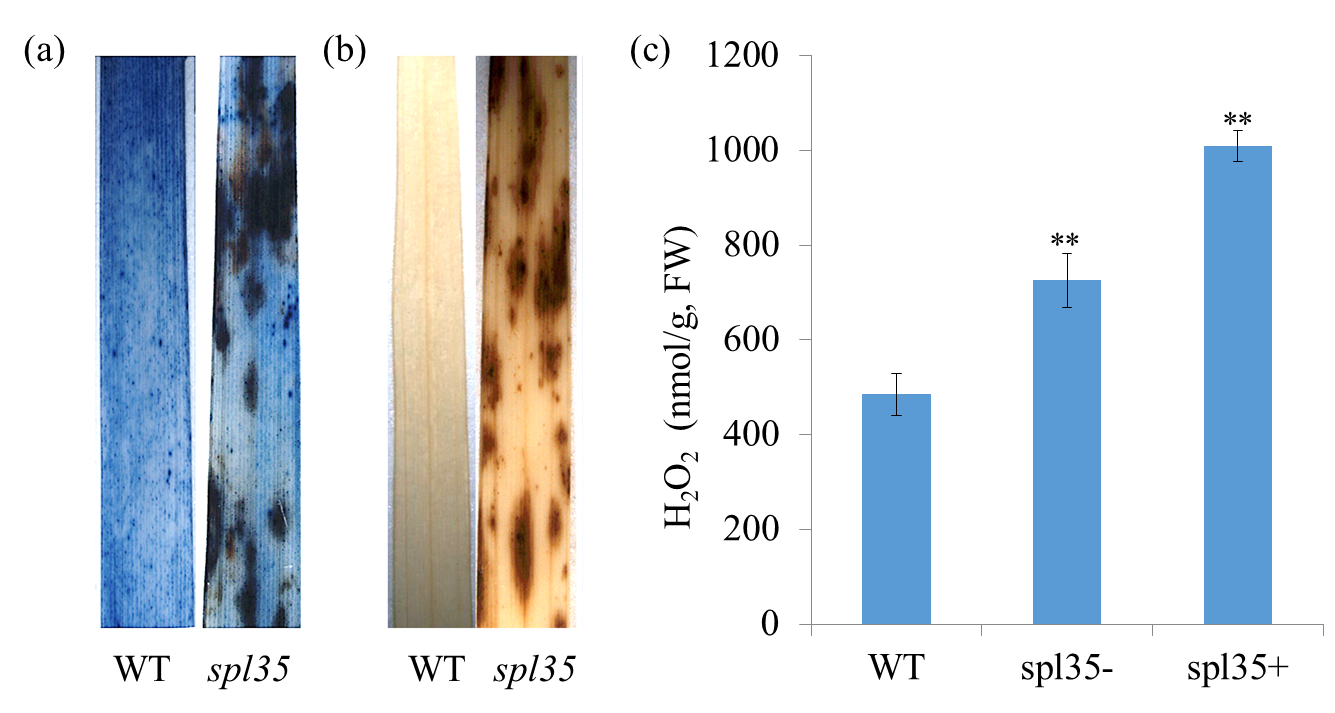

Supplement: Supplementary file 2 — Figure S2 The expression of histochemical markers and measurement of H2O2 in wild‐type (WT) and spl35 mutant. [file PBI-17-1679-s014.tif]

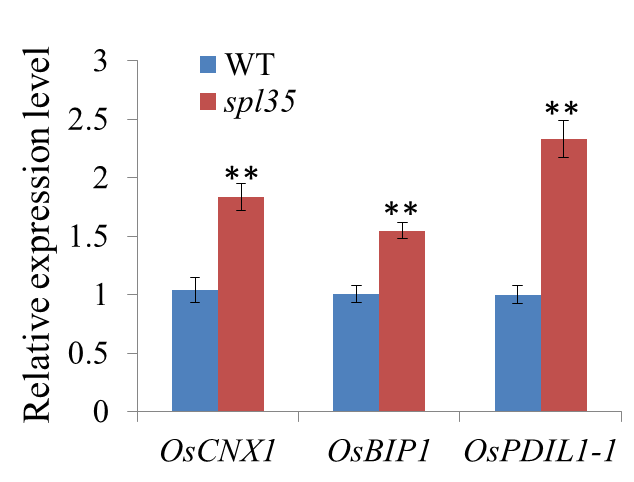

Supplement: Supplementary file 3 — Figure S3 Expression analysis of three endoplasmic reticulum (ER) chaperone genes in the wild‐type (WT) and spl35 plants by quantitative real‐time PCR (qRT‐PCR). [file PBI-17-1679-s013.tif]

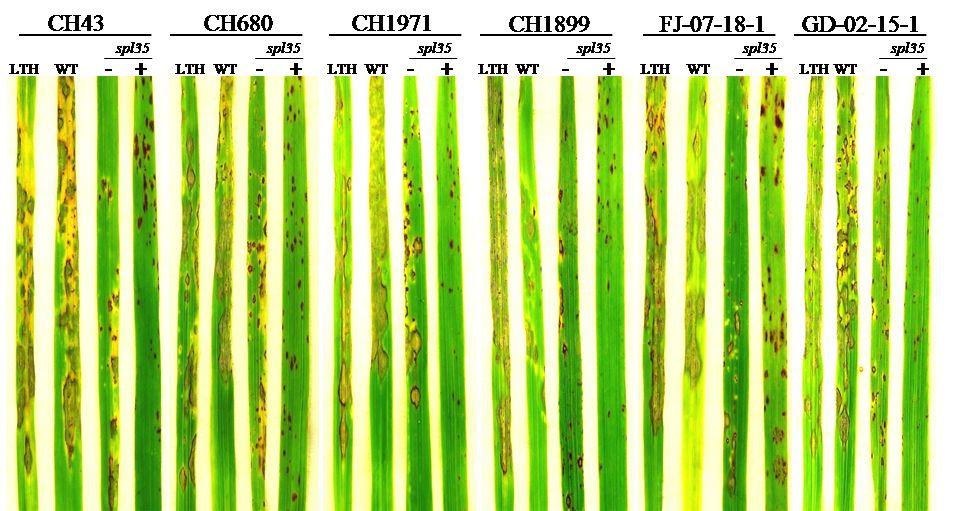

Supplement: Supplementary file 4 — Figure S4 Disease reactions of the wild‐type (WT) and spl35 mutant to six Maganaporthe oryzae isolates. [file PBI-17-1679-s017.tif]

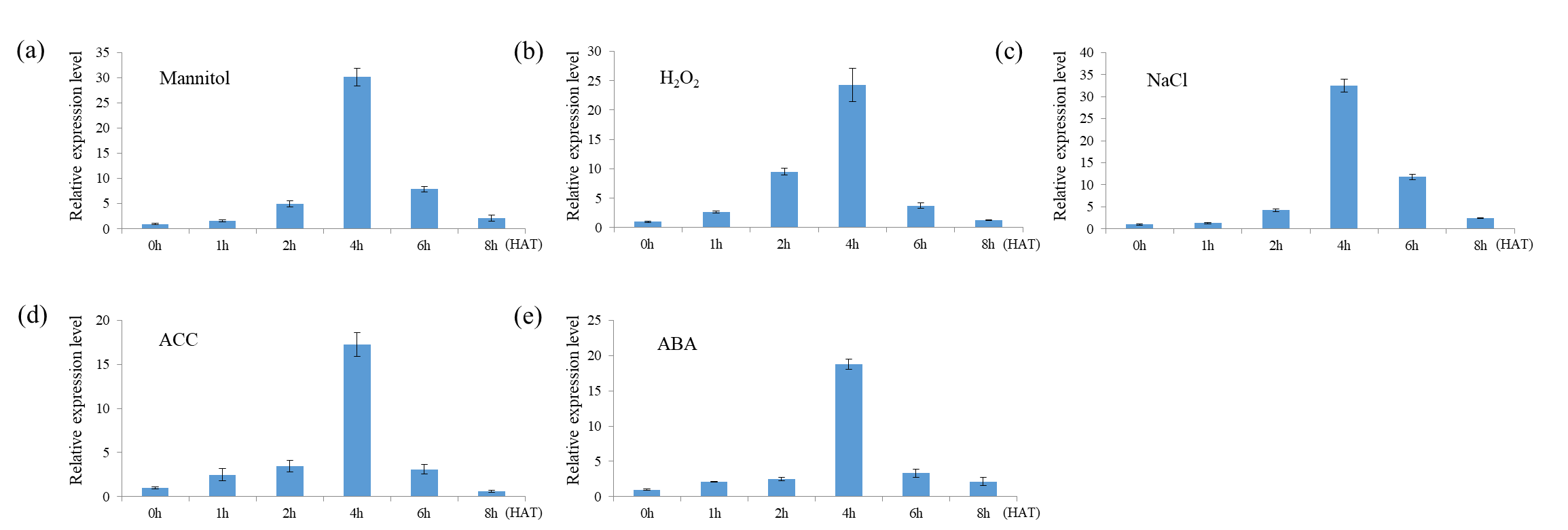

Supplement: Supplementary file 5 — Figure S5 Expression of SPL35 in response to different abiotic treatments. [file PBI-17-1679-s018.tif]

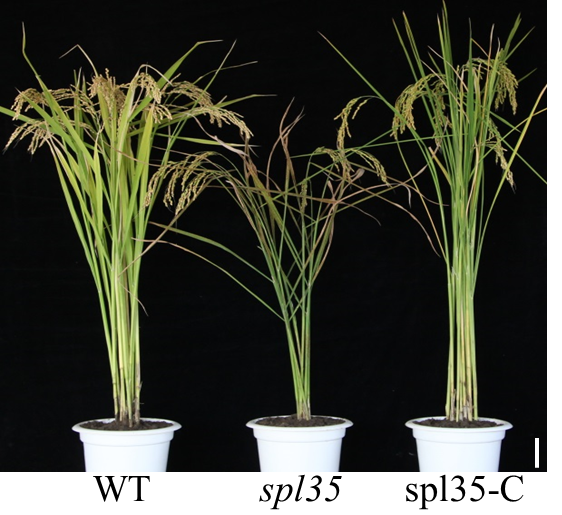

Supplement: Supplementary file 6 — Figure S6 Complementation assays of SPL35 transgenic plants. [file PBI-17-1679-s001.tif]

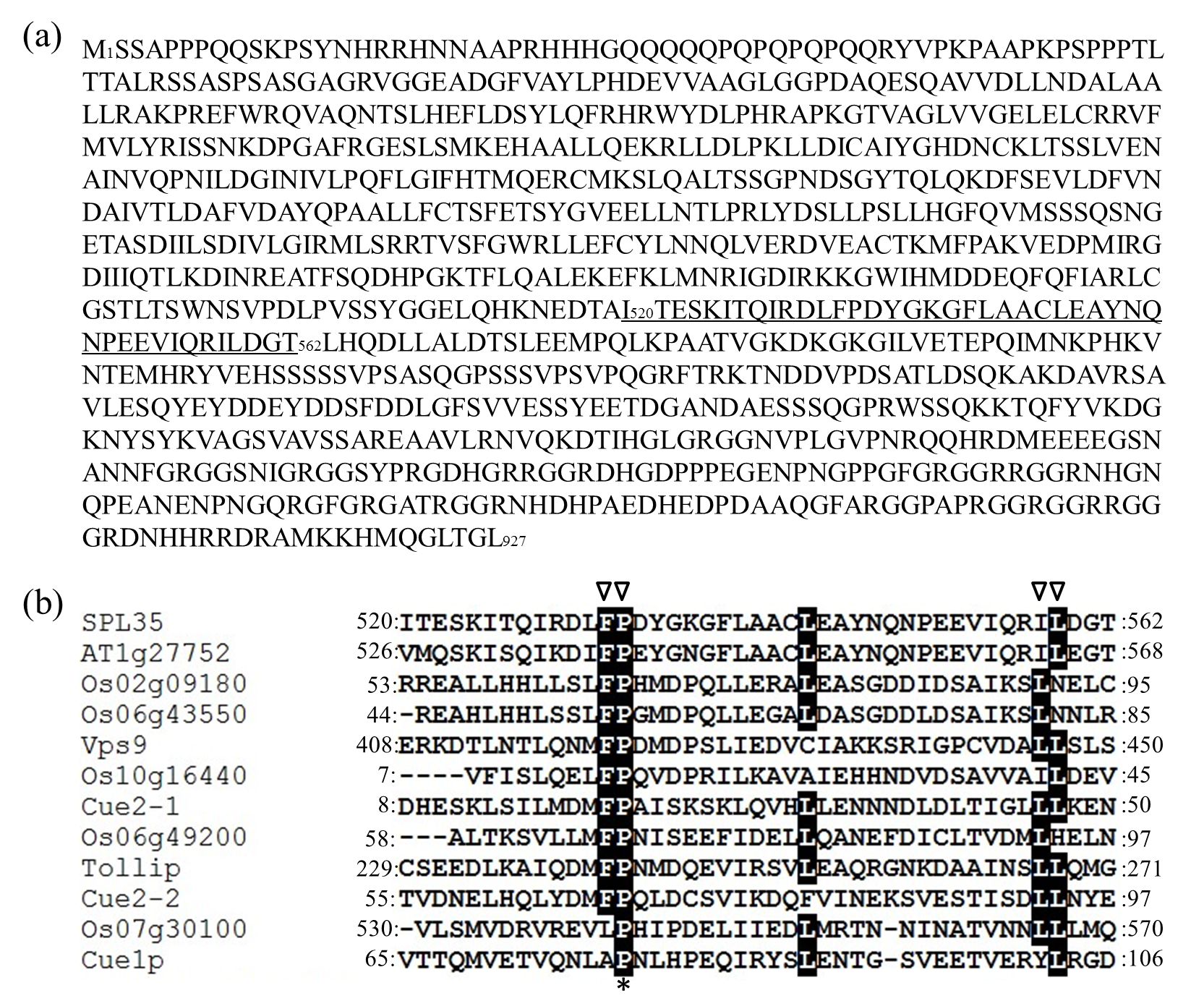

Supplement: Supplementary file 7 — Figure S7 SPL35 encodes a CUE domain‐containing protein. [file PBI-17-1679-s002.tif]

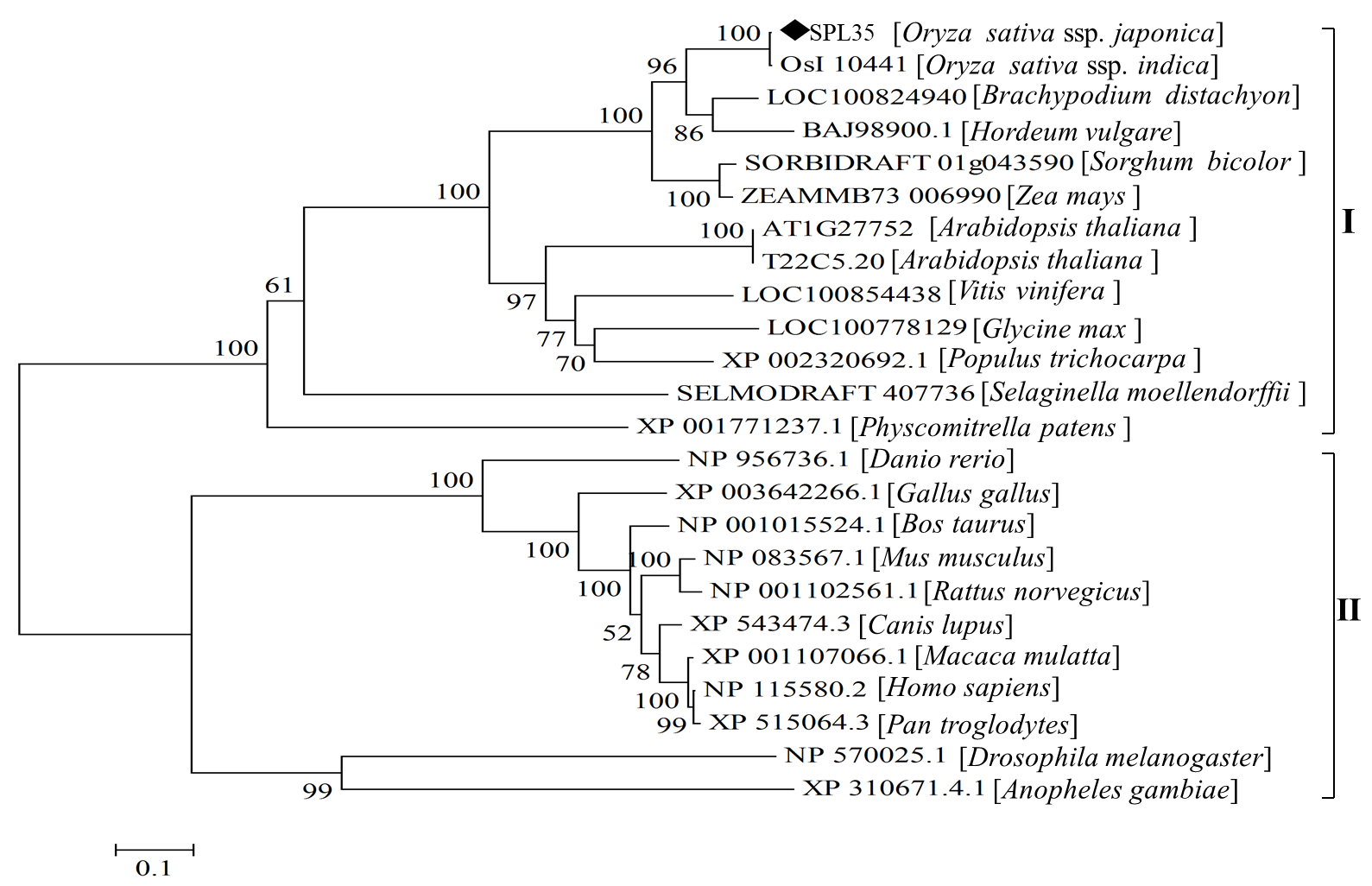

Supplement: Supplementary file 8 — Figure S8 Phylogenetic analysis of SPL35 with other homologues. [file PBI-17-1679-s003.tif]

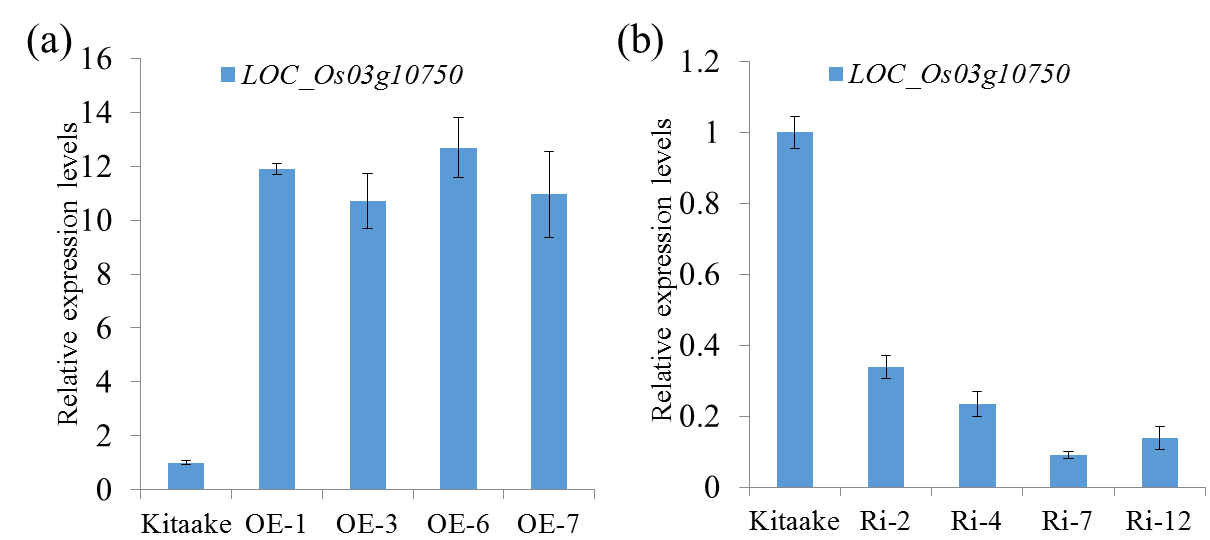

Supplement: Supplementary file 9 — Figure S9 Transcription levels of the LOC_Os03g10750 gene in different overexpressed (OE) or RNAi (Ri) transgenic lines (T1) detected by qRT‐PCR. [file PBI-17-1679-s004.tif]

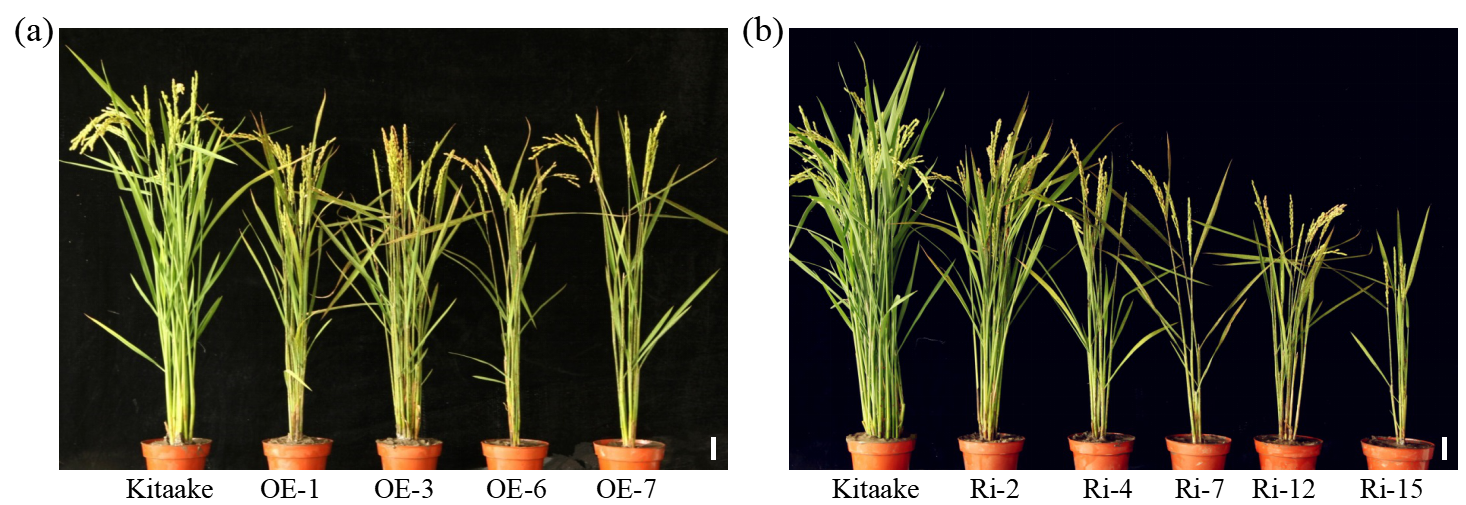

Supplement: Supplementary file 10 — Figure S10 Morphology of LOC_Os03g10750‐overexpressed (OE) and ‐RNAi (Ri) transgenic lines (T1) in Kitaake background under field conditions. Scale bars, 5.0 cm. [file PBI-17-1679-s005.tif]

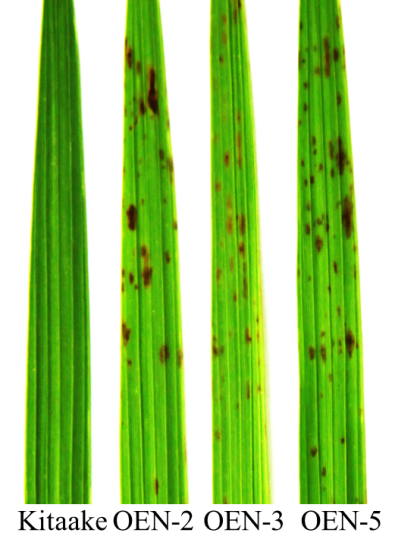

Supplement: Supplementary file 11 — Figure S11 Lesion mimics on the flag leaves of the transgenic lines (T1) overexpressing the N‐terminal 1‐568 amino acids of SPL35 in the cv. Kitaake (wild‐type) background OEN‐2, 3 and 5 represent the leaves of pSpl351‐568‐OE transgenic T1 lines in cv. Kitaake background. [file PBI-17-1679-s006.tif]

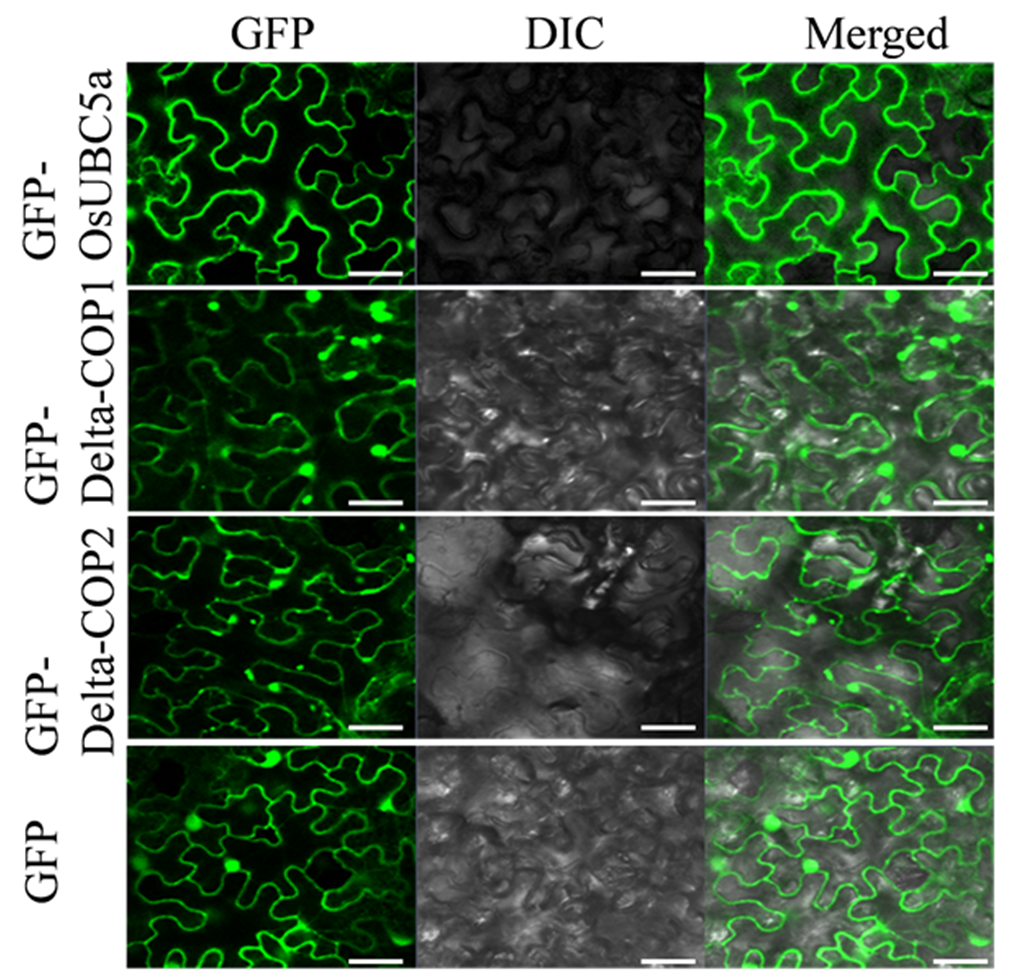

Supplement: Supplementary file 12 — Figure S12 Subcellular localization of GFP‐OsUBC5a, GFP‐Delta‐COP1 and GFP‐Delta‐COP2 fusion proteins in Nicotiana benthamiana leaf epidermal cells. GFP protein itself is distributed throughout the nucleus and cytoplasm (fourth panels). GFP, green fluorescent protein; DIC, differential interference contrast. Scale bars, 10 µm. [file PBI-17-1679-s007.tif]

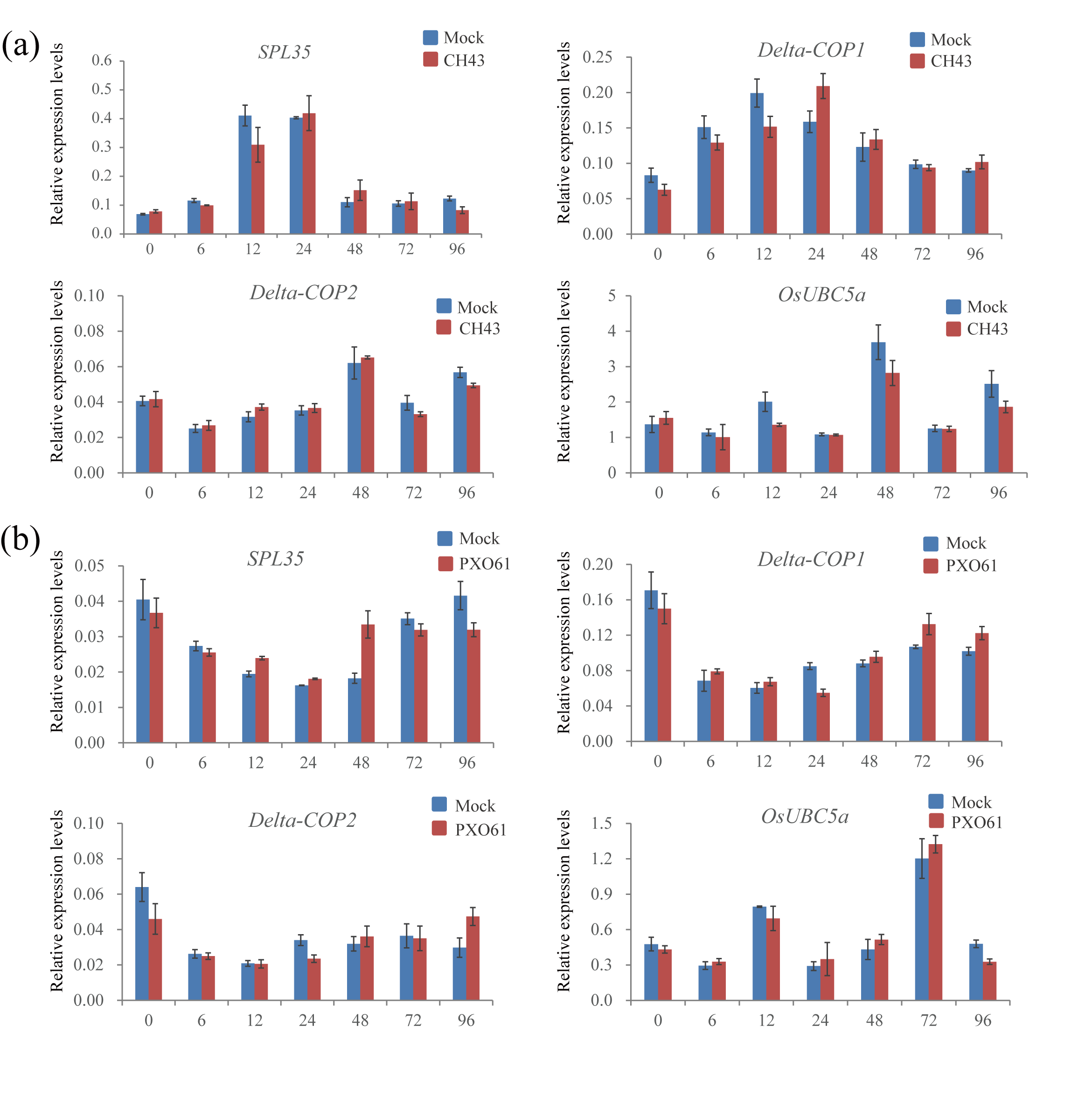

Supplement: Supplementary file 13 — Figure S13 qRT‐PCR analysis of transcript levels of SPL35, OsUBC5a, Delta‐COP1 and Delta‐COP2 during Maganaporthe oryzae (M. oryzae) and Xanthomonas oryzae pv. oryzae (Xoo) infections. [file PBI-17-1679-s008.tif]

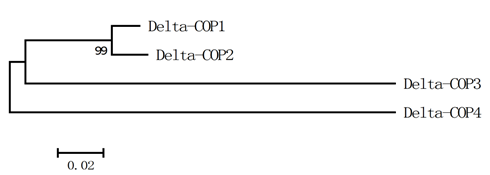

Supplement: Supplementary file 14 — Figure S14 The relationship of four coatomer subunit delta proteins in rice by unrooted phylogenetic tree analysis. [file PBI-17-1679-s009.tif]
