# Supplementary material for: Disruption of gene SPL35, encoding a novel CUE domain‐containing protein, leads to cell death and enhanced disease response in rice
Source: Plant Biotechnol J. 2019 Mar 5;17(8):1679–93. doi: 10.1111/pbi.13093 (PMC6662554; doi:10.1111/pbi.13093)
Supplement: Supplementary file 16 — Table S2 Comparison of agronomic traits between spl35 mutant and its wild‐type (WT, cv. Kinmaze). [file PBI-17-1679-s011.docx]

Table S2 Comparison of agronomic traits between the wild-type (WT, cv. Kinmaze) and *spl35* mutant

| **Trait** | **WT** | ***spl35*** |
| --- | --- | --- |
| Heading (days) | 116.3 ± 1.53 | 115.7 ± 1.15 |
| Plant height (cm) | 109.30 ± 2.50 | 96.85 ± 1.56^**^ |
| Panicle length (cm) | 21.52 ± 1.69 | 20.75 ± 1.09 |
| Number of effective panicles | 19 ± 2.71 | 15 ± 2.83^*^ |
| Number of spikelets per panicle | 162.8 ± 11.75 | 122.3 ± 8.12^**^ |
| Number of filled grains per panicle | 150.4 ± 11.20 | 89.9 ± 7.64^**^ |
| Seed setting rate (%) | 92.37 ± 0.75 | 73.61 ± 5.59^**^ |
| 1000-grain weight (g) | 2.26 ± 0.12 | 2.05 ± 0.02^**^ |

* and **, significantly different at P = 0.05 and P = 0.01, respectively.
