# Supplementary material for: Disruption of gene SPL35, encoding a novel CUE domain‐containing protein, leads to cell death and enhanced disease response in rice
Source: Plant Biotechnol J. 2019 Mar 5;17(8):1679–93. doi: 10.1111/pbi.13093 (PMC6662554; doi:10.1111/pbi.13093)
Supplement: Supplementary file 17 — Table S3 Disease reactions of the spl35 mutant to six M. oryzae isolates. [file PBI-17-1679-s012.docx]

Table S3 Disease reactions of the *spl35* mutant to six *M. oryzae* isolates

| Genotype | Blast isolate | | | | | |
| --- | --- | --- | --- | --- | --- | --- |
|  | CH43 | CH680 | CH1971 | CH1899 | FJ07-18-1 | GD02-15-1-1 |
| LTH | S | S | S | S | S | S |
| WT | S | S | S | S | S | S |
| *spl35-* | MS^a^ | MR^a^ | MS | MR | MS | MR |
| *spl35+* | R^d^ | R | R | R | R | R |

S, susceptible; MS, moderately susceptible; MR, moderately resistant; R, resistant. ^a^ The variable MS and MR responses were not considered significantly different.
