# Supplementary material for: Disruption of gene SPL35, encoding a novel CUE domain‐containing protein, leads to cell death and enhanced disease response in rice
Source: Plant Biotechnol J. 2019 Mar 5;17(8):1679–93. doi: 10.1111/pbi.13093 (PMC6662554; doi:10.1111/pbi.13093)
Supplement: Supplementary file 18 — Table S4 Genetic analysis of the spl35 mutant. [file PBI-17-1679-s016.docx]

Table S4 Genetic analysis of the *spl35* mutant

| Generation | No. of normal plants (AA/Aa) | No. of lesion mimic plants (aa) | Expected ratio | χ^2^_0.05_ | P_2df_ value |
| --- | --- | --- | --- | --- | --- |
| T_1_ | 4/9^a^ | 3 | 1:2:1 | 0.375 | 0.829 |
| T_2_ | 8/22^a^ | 12 | 1:2:1 | 2.762 | 0.251 |
| F_2_ (*spl35*/93-11) | 168 | 50 | 3:1 | 0.391 | 0.532 |

^a^ Heterozygous plants were identified using the hygronmycin B marker (HyrF/R).
